# β-SNAP activity in the outer segment growth period is critical for preventing BNip1-dependent apoptosis in zebrafish photoreceptors

**DOI:** 10.1038/s41598-020-74360-x

**Published:** 2020-10-15

**Authors:** Yuko Nishiwaki, Ichiro Masai

**Affiliations:** Developmental Neurobiology Unit, Okinawa Institute of Science and Technology Graduate University, 1919-1 Tancha, Onna, Okinawa 904-0495 Japan

**Keywords:** Disease model, Cell death in the nervous system, Apoptosis, Genetics

## Abstract

BNip1, which functions as a t-SNARE component of the syntaxin18 complex, is localized on the ER membrane and regulates retrograde transport from Golgi to the ER. BNip1 also has a BH3 domain, which generally releases pro-apoptotic proteins from Bcl2-mediated inhibition. Previously we reported that retinal photoreceptors undergo BNip1-dependent apoptosis in zebrafish *β-snap1* mutants. Here, we investigated physiological roles of BNip1-dependent photoreceptor apoptosis. First, we examined the spatio-temporal profile of photoreceptor apoptosis in *β-snap1* mutants, and found that apoptosis occurs only during a small developmental window, 2–4 days-post-fertilization (dpf), in which an apical photoreceptive membrane structure, called the outer segment (OS), grows rapidly. Transient expression of β-SNAP1 during this OS growing period prevents photoreceptor apoptosis in *β-snap1* mutants, enabling cone to survive until at least 21 dpf. These observations suggest that BNip1-mediated apoptosis is linked to excessive activation of vesicular transport associated with rapid growth of the OS. Consistently, knockdown of Ift88 and Kif3b, which inhibits protein transport to the OS, rescued photoreceptor apoptosis in *β-snap1* mutants. Treatment with rapamycin, which inhibits protein synthesis via the mTOR pathway, also rescued photoreceptor apoptosis in *β-snap1* mutants. These data suggest that BNip1 performs risk assessment to detect excessive vesicular transport in photoreceptors.

## Introduction

More than a hundred genes associated with inherited photoreceptor degeneration have been identified in humans (See the homepage of the retinal information network: https://sph.uth.edu/retnet/)^1^. These genes encode proteins with great functional diversity, and are involved in phototransduction, retinol metabolism, scaffolding of the apical photoreceptive membrane organelle known as the outer segment (OS), and transport of proteins to the OS through the connecting cilium. However, it remains to be seen how dysfunction of these proteins triggers photoreceptor cell death, especially apoptosis.

Previously, we screened zebrafish mutants that show photoreceptor degeneration, and identified zebrafish *corona* (*coa*) mutants^2^. In *coa* mutants, photoreceptors are produced normally after 2 days-post-fertilization (dpf), but rapidly undergo apoptosis and are completely eliminated by 6 dpf. The *coa* mutant gene encodes β-soluble N-ethyl-maleimide-sensitive factor attachment protein 1 (β-SNAP1). SNAP regulates the fusion process of transport vesicles to the target membrane in eukaryotes^3^. Three forms of SNAPs have been identified in mammals: α-, β-, and γ-SNAP. β-SNAP is expressed specifically in the brain and shows high amino acid similarity to α-SNAP^4^. SNAP receptors (SNAREs) are another important factor^5^, and are classified into two groups: vesicle-membrane SNAREs (v-SNAREs) and target membrane SNAREs (t-SNAREs). Three t-SNARE components bind to form acceptor complexes on target membranes, which interact with v-SNAREs of transport vesicles to initiate docking and fusion of transport vesicles to target membranes. The acceptor complex and v-SNARE form a cis-SNARE complex on fused membranes. After that, SNAP binds the cis-SNARE complex and recruits *N*-ethyl-maleimide-sensitive factor (NSF). NSF belongs to the AAA (ATPase-associated diverse cellular activity) protein family^6^. Using energy from ATP hydrolysis, NSF disassembles the cis-SNARE complex to recycle the SNAREs^7^. Since vesicular fusion is compromised in the absence of β-SNAP1 activity, *coa* mutants provide a good model to investigate how vesicular fusion defects cause photoreceptor apoptosis. Our previous study showed that BNip1 mediates photoreceptor apoptosis in *coa* mutants^2^.

BNip1 was identified as a protein that interacts with anti-apoptotic adenovirus E1B 19 kDa protein^8^. BNip1 has a Bcl2 homology domain 3 (BH3), which generally binds to an anti-apoptotic protein, Bcl2, and releases a proapoptotic protein, Bax, from Bcl2-mediated inhibition. Indeed, BNip1 overexpression induces moderate apoptosis in human cell cultures^9,10^. Interestingly, BNip1 has a SNARE domain, so it functions as a t-SNARE component of the syntaxin-18 complex. The syntaxin-18 complex consists of three t-SNARE components, syntaxin-18, BNip1, Unconventional SNARE in the ER1 (Use1), and one v-SNARE component, Sec22b, and regulates retrograde vesicular transport from Golgi bodies to the endoplasmic reticulum (ER)^9^. After fusion of retrogradely transported vesicles on the ER membrane, the syntaxin-18 cis-SNARE complex is generated. This complex is normally disassembled by SNAP and NSF. However, in *coa* mutants, the syntaxin-18 cis-SNARE complex fails to be disassembled for lack of β-SNAP activity, resulting in activation of BNip1, BH3-dependent apoptosis in photoreceptors. A possible mechanism underlying BNip1-dependent apoptosis is that BNip1 pro-apoptotic activity is suppressed by intramolecular binding of the N-terminal coiled-coil domain to the BH3 domain in the monomeric state; however, when BNip1 forms the syntaxin-18 cis-SNARE complex, the BNip1 BH3 domain interacts with Bcl2, resulting in Bax-dependent apoptosis. In this context, BNip1 monitors reduction of β-SNAP activity and activates apoptosis when β-SNAP activity is decreased.

However, there are several questions that have not been addressed. For example, what physiological situations decrease β-SNAP activity in vivo? Since the absence of β-SNAP activity compromises recycling of all SNARE proteins, the intracellular protein transport system may eventually become arrested in *coa* mutants, which is likely to activate the ER-stress response^11^. However, it is unknown whether the ER-stress response is activated in *coa* mutants. If so, how are both the ER-stress response and BNip1-mediated apoptosis coordinated in *coa* mutants? To answer these questions, we investigated physiological roles of BNip1-dependent photoreceptor apoptosis. We found that photoreceptor apoptosis occurs in *coa* mutants during a short developmental window from 2 to 4 dpf, during which the OS rapidly grows. Transient expression of β-SNAP1 in *coa* mutants during this OS growing period prevents significant photoreceptor apoptosis, enabling cone to survive until 21dpf. This suggests that BNip1-mediated apoptosis is associated with excessive activation of vesicular transport associated with rapid growth of the OS. One interesting scenario is that increased vesicular transport traps β-SNAP molecules on vesicular fusion sites, subsequently decreasing the relative contribution of β-SNAP to vesicular fusion in the ER, and accumulating syntaxin-18 cis-SNARE complexes on the ER membrane. Indeed, knockdown of Ift88 and Kif3b, which inhibits protein transport to the OS^12,13^, or rapamycin treatment, which inhibits protein synthesis^14^, rescued photoreceptor apoptosis in *coa* mutants. These data suggest that BNip1 performs risk assessment that detects excessive activation of vesicular transport in photoreceptors.

## Results

### ER-targeted Bcl2 effectively prevents photoreceptor apoptosis in *coa* mutants

**Figure 1:**
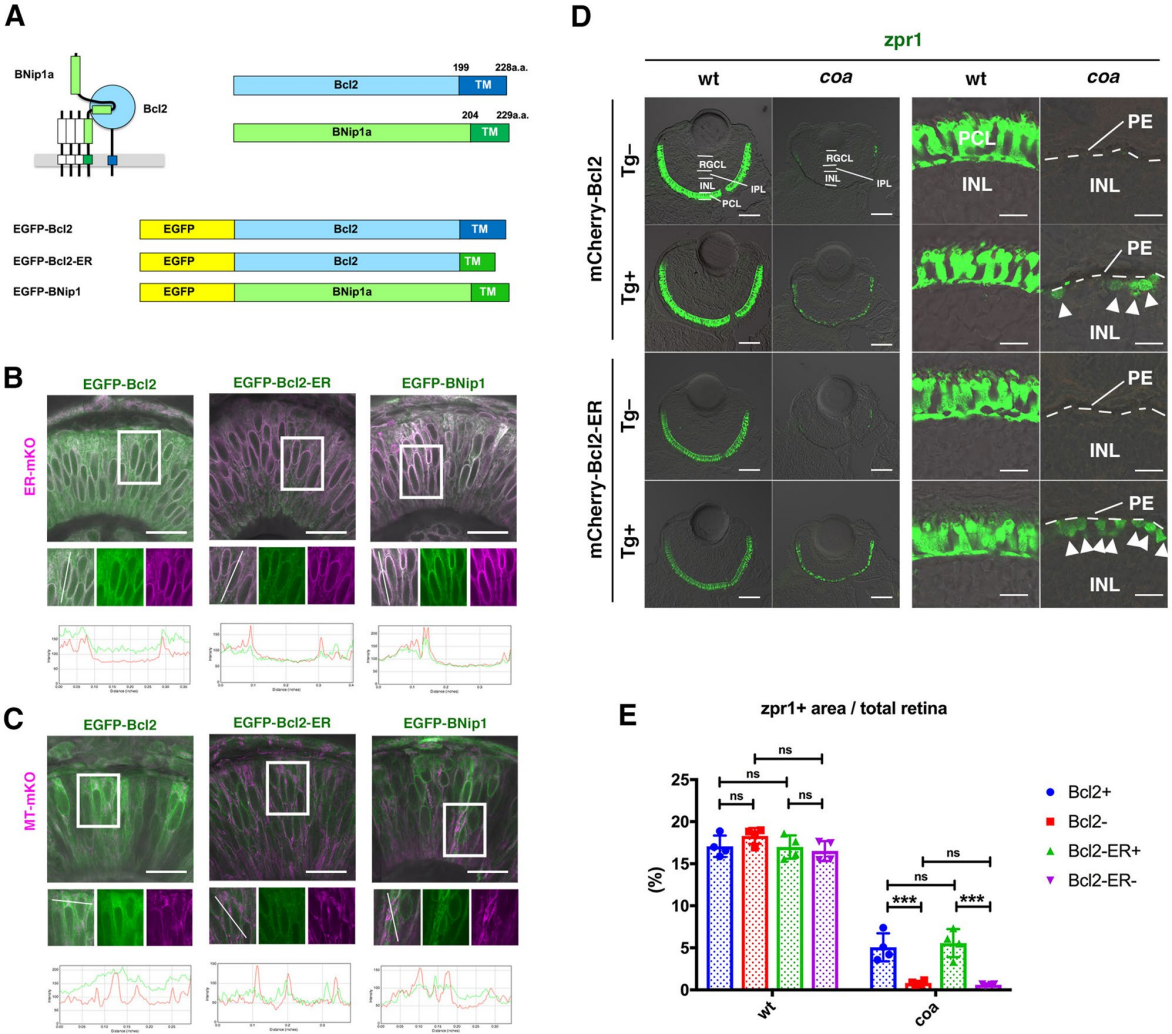
Bcl2-ER significantly rescues photoreceptor apoptosis in *coa* mutants. (**A**) DNA construction of EGFP-tagged Bcl2, EGFP-tagged Bcl2-ER, and EGFP-tagged BNip1. EGFP-tagged Bcl2-ER was generated by replacement of the Bcl2 TM domain with a BNip1 TM domain. (**B**) Retinas injected with mRNA encoding ER-mKO (magenta) and either mRNA encoding EGFP-tagged Bcl2, EGFP-tagged Bcl2-ER, or EGFP-tagged BNip1 (green). Fluorescent images shown in squares of upper panels, and their green and magenta channels (middle panels). Bottom histograms indicate spatial profiles of EGFP and mKO signals along the line shown in the middle panels. Like EGFP-tagged BNip1, the spatial profile of EGFP-tagged Bcl2-ER signals correlates with that of ER-mKO signals, whereas EGFP-tagged Bcl2 signals are not correlated. Scale: 20 μm. (**C**) Retinas injected with mRNA encoding MT-mKO (magenta) and either mRNA encoding EGFP-tagged Bcl2, EGFP-tagged Bcl2-ER, or EGFP-tagged BNip1 (green). Fluorescent images shown in squares of upper panels, and their green and magenta channels (middle panels). Bottom histograms indicate spatial profiles of EGFP and mKO signals along the line shown in the middle panels. Like EGFP-tagged BNip1, peaks of EGFP-tagged Bcl2-ER signals do not match those of MT-mKO signals, whereas EGFP-tagged Bcl2 signals are broader than MT-mKO signals. Scale: 20 μm. (**D**) Retinas of wild-type and *coa* mutant embryos combined with transgenic lines *Tg[hs:mCherry-tagged Bcl2]* or *Tg[hs:mCherry-tagged Bcl2-ER]*. Tg+ and Tg− indicate transgenic and non-transgenic embryos, respectively. Green color indicates zpr1 antibody signals, which label double-cone photoreceptors. The retinal ganglion cell layer (RGCL), inner plexiform layer (IPL), INL and photoreceptor cell layer (PCL) are shown. Right two columns indicate higher magnification of the PCL shown in the left two columns. The interface between pigmented epithelium (PE) and the neural retina is shown as a white, dotted line. Arrowheads indicate rescued photoreceptors. Scale: 50 μm (left two columns) and 10 μm (right two columns). (**E**) Percentage of zpr1-positive area relative to total retinal area. Both Bcl2 and Bcl2-ER partially, but significantly, increase the zpr1-positive fraction in *coa* mutants, suggesting that as with Bcl2, Bcl2-ER effectively rescues photoreceptor apoptosis in *coa* mutants. Means ± SD. Two-way ANOVA with the Tukey multiple comparison test. ***p < 0.005.

Our previous study on zebrafish *coa* mutants suggested a model in which disassembly failure of syntaxin-18 cis-SNARE complexes facilitates BNip1′s interaction with Bcl2 through its BH3 domain on the ER membrane, sequestering Bcl2 from Bax (Fig. 1A)^2^. To confirm this model, we examined whether BNip1′s pro-apoptotic activity is initiated on the ER membrane. Since zebrafish BNip1 interacts with Bcl2 in vitro^2^, we designed ER-targeted Bcl2 (Bcl2-ER), in which Bcl2 transmembrane (TM) is replaced with BNip1 TM. Furthermore, N-termini of Bcl2-ER were tagged with a fluorescent protein, either EGFP or mCherry (Fig. 1A). We confirmed that EGFP-Bcl2 was located in cytoplasm, ER, and mitochondria, whereas EGFP-Bcl2-ER was predominantly found in ER (Fig. 1B, C).

Next, we generated a zebrafish transgenic line, *Tg[hs:mCherry-Bcl2-ER]* that expresses mCherry-Bcl2-ER under control of the heat shock promoter, and combined it with *coa* mutants. We conducted TUNEL and zpr1 antibody labeling to evaluate photoreceptor apoptosis and survival. As we showed previously^2^, overexpression of Bcl2 by heat shock treatment at 36/48/60/72/84 h-post fertilization (hpf) significantly rescues photoreceptor apoptosis in *coa* mutants at 96 hpf (Fig. 1D, S1A). Overexpression of Bcl2-ER by heat shock treatment at 36/48/60/72/84 hpf also significantly rescues photoreceptor apoptosis in *coa* mutants at a similar level of Bcl2 overexpression (Fig. 1D, E, S1A, S1B). Thus, apoptotic activity of BNip1 is activated on the ER membrane and overexpression of Bcl2 on the ER membrane effectively suppresses its apoptotic activity.

To exclude the possibility that overexpression of Bcl2-ER by the heat shock promoter might cause Bcl2-ER to leach out of the ER, we used another zebrafish mutant, *pinball eyes* (*piy*), which carries a missense mutation in the *DNA primase subunit 1* (*prim1*) gene. In this mutant, DNA replication stress is abnormally activated, leading to activation of p53-dependent apoptosis in retinal neurons^15^. In *piy* mutants, p53 activates downstream BH3 proteins such as PUMA, which directly suppress Bcl2 in cytoplasm or on mitochondrial membranes, leading to activation of Bax on mitochondrial membranes^15^. We combined *piy* mutants with transgenic lines *Tg[hs:mCherry-Bcl2]* or *Tg[hs:mCherry-Bcl2-ER]*, conducted heat shock treatment at 36/48 hpf and examined retinal apoptosis at 60 hpf. In the *piy* mutant without the transgene, almost all retinal neurons underwent apoptosis by 60 hpf (Fig. S1C). On the other hand, retinal apoptosis in *piy* mutants was suppressed by expression of mCherry-Bcl2, but less effectively by mCherry-Bcl2-ER (Fig. S1C, S1D), suggesting that Bcl2-ER is specifically located on the ER membrane. These observations support the current model that lack of β-SNAP activity causes accumulation of the syntaxin-18 cis-SNARE complex, which facilitates the interaction between the BNip1 BH3 domain and Bcl2 on the ER membrane in *coa* mutant photoreceptors.

### Photoreceptor apoptosis occurs during the OS growth period in *coa* mutants

**Figure 2:**
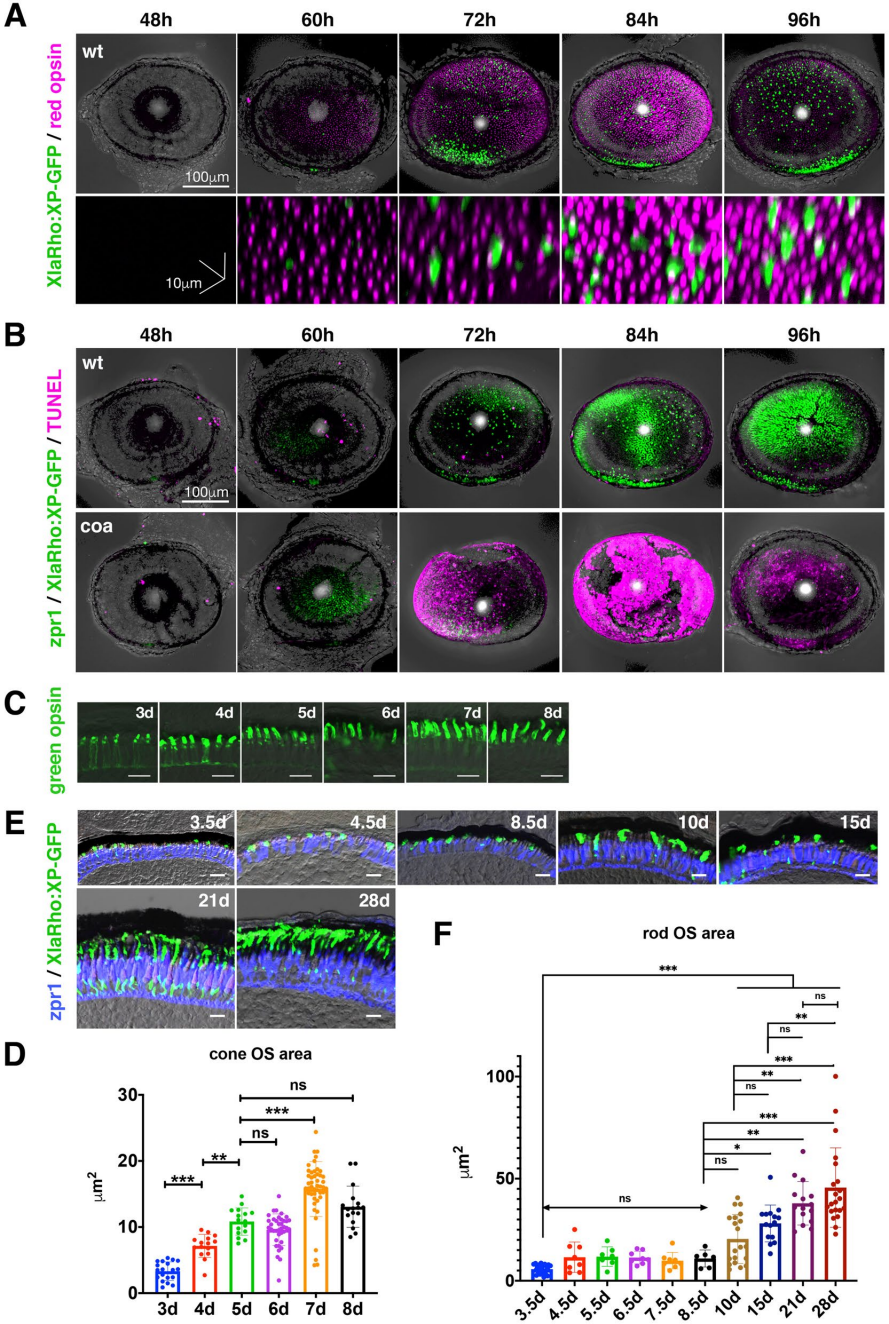
Apoptosis occurs during OS growth stages from 60 to 96 hpf. (**A**) Whole mount retinas of zebrafish transgenic embryos, *Tg[XlaRho:XP-GFP]* (green), labelled with anti-red opsin antibody (magenta). Green and magenta colors indicate rod and cone photoreceptor OSs, respectively. There is no OS signal at 48 hpf. The OS appears at 60 hpf and progressively increases in size until 96 hpf. Scale:100 μm (upper) and 10 μm (bottom). (**B**) Whole mount retinas of zebrafish transgenic embryos, *Tg[XlaRho:XP-GFP]* (green), labelled with zpr1 antibody (green) and TUNEL (magenta). Upper and lower panels indicate wild-type and the *coa* mutant genetic background, respectively. In wild-type retinas, a few TUNEL-positive cells were observed at each stage. However, in *coa* mutants, dense TUNEL signals appear at 72 hpf, spread to the entire retina by 84 hpf and disappear by 96 hpf. The lack of zpr1 and XP-GFP signals in *coa* mutants at 96 hpf suggest that almost all photoreceptors are eliminated by 96 hpf. Thus, almost all photoreceptors undergo apoptosis from 60 to 96 hpf. Scale: 100 μm. (**C**) Sectioned images of the photoreceptor cell layer labelled with anti-green opsin antibody (green) at 3, 4, 5, 6, 7 and 8 dpf. The cone OS grows during development. Scale: 10 μm. (**D**) Green cone OS size measured using section images shown in (C). The green cone OS increases linearly from 3 to 5 dpf, and then reaches a plateau. Means ± SD. One-way ANOVA with the Tukey multiple comparison test. **p < 0.01, ***p < 0.005. (**E**) Sectioned images of the photoreceptor cell layer labelled with fluorescent signals of *Tg[XlaRho:XP-GFP]* (green) at 3.5, 4.5, 8.5, 10, 15, 21, and 28 dpf. The cone photoreceptor cell layer is counterstained with zpr1 antibody (blue). The rod OS grows during development. Scale: 10 μm. (**F**) Rod OS size measured using section at 3.5, 4.5, 5.5, 6.5, 7.5, 8.5, 10, 15, 21, and 28 dpf. The rod OS initially increases until 4.5 dpf, is maintained as a plateau from 4.5 to 8.5 dpf, and again starts to grow after 8.5 until 28 dpf. One-way ANOVA with Dunnett’s multiple comparison test. *p < 0.05, **p < 0.01, ***p < 0.005.

Next, we examined photoreceptor apoptosis in *coa* mutants at developmental stages from 48 to 96 hpf. OSs of rods and cones were labeled with a transgene, *Tg[XlaRho:XP-GFP]*, which expresses GFP-tagged *Xenopus* Peripherin 2 under control of the *Xenopus rhodopsin* promoter^16^, and anti-red opsin antibody^17^, respectively. These rod and cone OS markers were first detected at 60 hpf in wild-type retinas. The size and density of OSs progressively increased from 60 to 96 hpf, suggesting that the OS actively grows from 48 to 96 hpf in zebrafish (Fig. 2A). zpr1 is a marker of double cone-type photoreceptors (red and green cones) in zebrafish^18^. TUNEL was applied to wild-type and *coa* mutant retinas and counterstained with *Tg[XlaRho:XP-GFP]* and zpr1 antibody. In *coa* mutants, photoreceptor apoptosis occurs after 60 hpf, and then promptly spreads into the whole retinal region until 84 hpf. Most photoreceptors were eliminated by 96 hpf (Fig. 2B). On the other hand, such concentrated apoptosis was not observed in wild type retinas. Thus, photoreceptor apoptosis occurs from 60 to 96 hpf in *coa* mutants when the OS grows rapidly.

In zebrafish, OS length in cones does not increase drastically after 8 dpf and it reaches adult OS size at 15 dpf, whereas the rod OS rapidly increases in size between 12 and 20 dpf and is maintained until the adult stage^19^. We evaluated the OS growth rate of rods and cones. Cone OS area was measured in wild-type retinal sections labeled with anti-green opsin antibody^20^, from 3 to 8 dpf (Fig. 2C). Cone OS size increased linearly from 3 to 5 dpf, and plateaued after 5 dpf, although small transient increases were observed at 7 dpf (Fig. 2D), which may result from a balance between synthesis of new OS and daily phagocytosis of old parts of the OS by pigmented epithelium. Next, rod OS area was measured in wild-type retinal sections labeled with fluorescent *Tg[XlaRho:XP-GFP]*, from 3.5 to 28 dpf (Fig. 2E). Since rod OS genesis is markedly enhanced in the ventral retina in zebrafish because of retinoic acid signaling^21,22^, we measured rod OS size in the dorsal retina. Rod OS size initially increased until 4.5 dpf, then plateaued around 10 μm^2^ from 4.5 to 8.5 dpf, and again started to increase until 28 dpf (Fig. 2F), indicating two phases for rod OS growth. Thus, rod and cone apoptosis occur in *coa* mutants during their initial OS growth from 2 to 4 dpf.

### Overexpression of β-SNAP1 during the OS growth period prevents photoreceptor apoptosis in ***coa*** mutants

**Figure 3:**
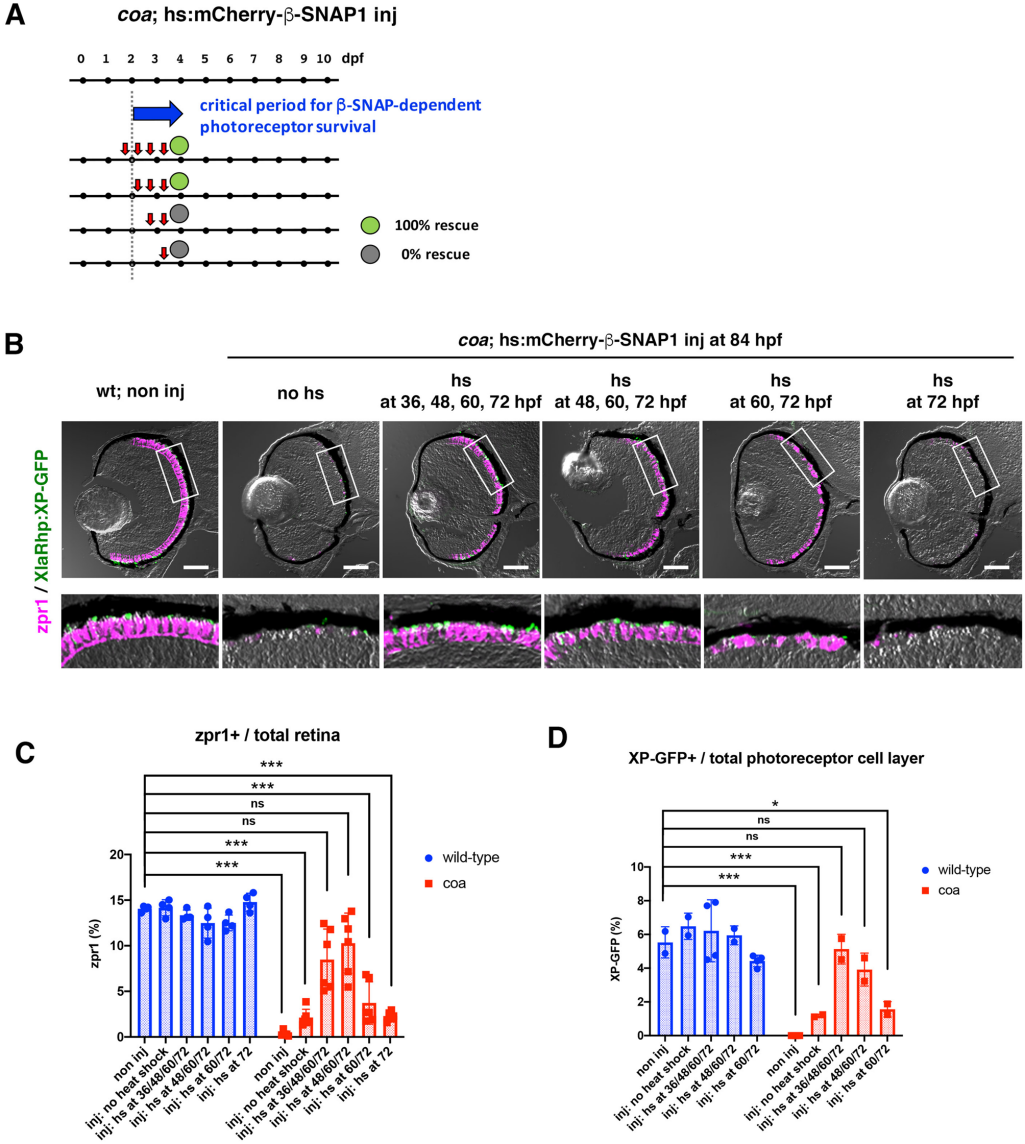
β-SNAP1 activity after 48 hpf is required for photoreceptor survival at 84 hpf. (**A**) Experimental design and results of heat-shock promoter-driven β-SNAP1 overexpression in *coa* mutants. Zebrafish embryos were injected with a DNA construct that expresses mCherry-tagged β-SNAP1 under control of the heat-shock promoter. Heat-shock treatment of injected embryos was performed at 36, 48, 60 and 72 hpf (red arrows), and photoreceptor apoptosis was examined at 84 hpf. Green or grey circles indicate rescue results for cone survival. (**B**) Retinas of wild-type, non-injected, control embryos and *coa* mutant embryos injected with a DNA construct encoding hs:mCherry-β-SNAP1 at the one-cell stage and heat-shocked four times at 36/48/60/72 hpf, three times at 48/60/72 hpf, two times at 60/72 hpf, once at 72 hpf, or no heat shock treatment. Cone and rod survival was confirmed by labeling with zpr1 antibody (magenta) and fluorescent signals of *Tg[XlaRho:XP-GFP]* (green). Three or four heat-shock treatments effectively, and two heat-shock treatments mildly, rescued photoreceptor degeneration in *coa* mutants. Scale: 50 μm. (**C**) Percent zpr1-positive area relative to total retinal area. Blue and red bars indicate wild-type and *coa* mutant embryos injected with DNA encoding hs:mCherry-β-SNAP1. Cone apoptosis was rescued by heat shock treatment at 36/48/60/72 hpf and 48/60/72 hpf, which shows an equivalent rescue level to wild type. Means ± SD. Two-way ANOVA with the Tukey multiple comparison test. ***p < 0.005. (**D**) Percent XP-GFP-positive area relative to the total area of the photoreceptor cell layer. Blue and red bars indicate wild-type and *coa* mutant embryos injected with DNA encoding hs:mCherry-β-SNAP1. Rod apoptosis was rescued by heat shock treatment at 36/48/60/72 hpf and 48/60/72 hpf, which shows an equivalent rescue level to wild type. Means ± SD. Two-way ANOVA with Sidak’s multiple comparison test. *p < 0.05, ***p < 0.005.

Since OS growth depends on synthesis of proteins and lipids and their transport to the OS, it is likely that photoreceptor apoptosis is linked to excessive activation of intracellular vesicular transport. We determined the timing of the critical period of β-SNAP1 activity to prevent photoreceptor apoptosis. A DNA construct that expresses N-terminal mCherry-tagged β-SNAP1 under control of the heat shock promoter was injected into zebrafish eggs at the one-cell stage. We confirmed that a one-hour pulse of heat shock treatment at 48 hpf induced mCherry expression at 60 hpf, which declined to 50% at 72 hpf and disappeared at 84 hpf (Fig. S2). Heat-shock treatment at 60 hpf induced mCherry expression at 72 and 84 hpf (Fig. S2C). Heat-shock treatment at 72 hpf induced mCherry expression at 84 hpf (Fig. S2C). Thus, a one-hour pulse of heat shock treatment introduces ectopic β-SNAP1 expression for at least 24 h. Next, we injected the DNA construct into *coa* mutants, and repeated the heat shock treatment at 12-h intervals from 36 to 72 hpf at four different starting points (36, 48, 60, 72 hpf). Cone and rod survival was evaluated at 84 hpf by labeling with zpr1 antibody and fluorescent signals of *Tg[XlaRho:XP-GFP]*, respectively (Fig. 3A). Neither heat shock treatment at 72 nor 60/72 hpf recovered cone and rod survival in *coa* mutants at 84 hpf (Fig. 3B–D). However, either 4 heat-shock treatments at 36/48/60/72 hpf or 3 heat-shock treatments at 48/60/72 hpf effectively recovered cone and rod survival in *coa* mutants (Fig. 3B–D). These results suggest that β-SNAP1 activity after 48 hpf is required to prevent photoreceptor apoptosis in *coa* mutants at 84 hpf.

**Figure 4:**
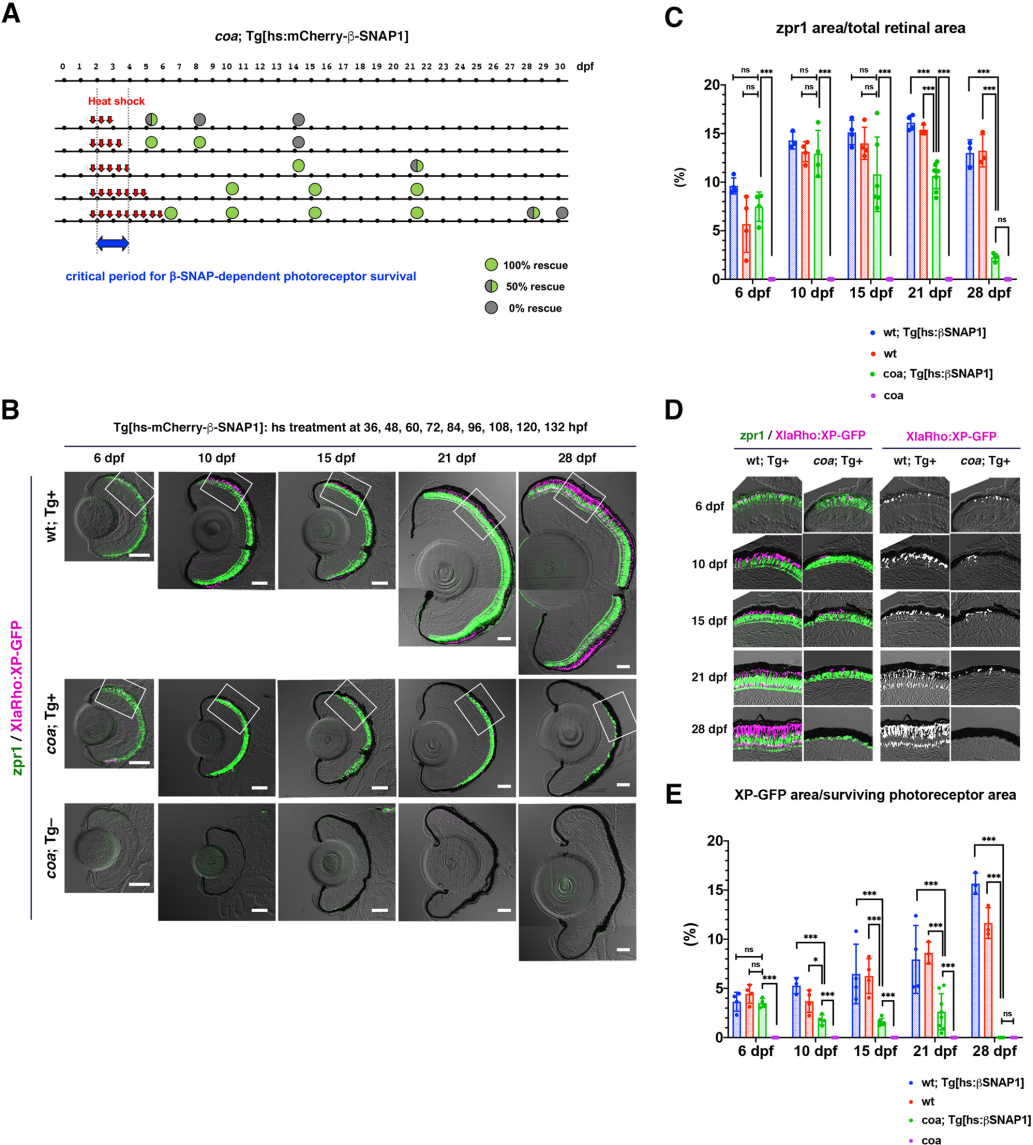
β-SNAP1 overexpression from 36 to 132 hpf is enough to maintain cones until 21 dpf but not rods in *coa* mutants. (**A**) Experimental design of β-SNAP1 overexpression in *coa* mutants. Heat treatments were applied at 12-h intervals from 36 to 132 hpf (red arrows). Green or grey circles indicate rescue results of cone survival and stage of analysis. The blue arrow indicates the critical period for β-SNAP1-dependent cone survival. (**B**) Retinas of *coa*; *Tg[XlaRho:XP-GFP]*; *Tg[hs:mCherry-β-SNAP1]* embryos or larvae that were heat-shocked from 36 to 132 hpf. Wild-type with *Tg[hs:mCherry-β-SNAP1]* and *coa* mutant without *Tg[hs:mCherry-β-SNAP1]* are positive and negative controls, respectively. Cone photoreceptors and rod OS were visualized by labeling with zpr1 antibody (green) and GFP signals from *Tg[XlaRho:XP:GFP]* (magenta), respectively. In *coa* mutants with *Tg[hs:mCherry-β-SNAP1]*, photoreceptors were maintained until 21 dpf, but degenerated at 28 dpf. Scale: 50 μm. (**C**) Percentage of zpr1-positive area relative to total retinal area. There was no significant difference in cone survival between wild-type embryos with/without *Tg[hs:mCherry-β-SNAP1]* and *coa* mutant embryos with *Tg[hs:mCherry-β-SNAP1]* at 6, 10, and 15 dpf. At 21 dpf, the fraction of surviving cones in *coa* mutants with *Tg[hs:mCherry-β-SNAP1]* started to decrease although it was still significantly higher than that of *coa* mutants. At 28 hpf, surviving cones markedly decreased in *coa* mutants with *Tg[hs:mCherry-β-SNAP1]* in a similar level to *coa* mutants. Means ± SD. Two-way ANOVA with the Tukey multiple comparison test, and Multiple t-test. ***p < 0.005. (**D**) Higher magnification of photoreceptor cell layers indicated by squares of panel (B). Magenta channel is shown in the right. The rod OS is increased in wild type during development. However, the rod OS in *coa* mutants was similar to that of wild type at 6 dpf, decreased at 10–21 dpf, and disappeared at 28 dpf. (**E**) Percentage of XP-GFP-positive area relative to total area of the photoreceptor cell layer. In wild type controls, the XP-GFP-positive fraction increases progressively during development. However, in *coa* mutant embryos with *Tg[hs:mCherry-β-SNAP1]*, the fraction is similar to that of wild type at 6 dpf, significantly decreased after 10 dpf and became 0% at 28 dpf. Means ± SD. Two-way ANOVA with Sidak’s multiple comparison test, and Multiple t-test. *p < 0.05, ***p < 0.005.

Next, we generated a zebrafish transgenic line, *Tg[hs:mCherry-β-SNAP1]*, which expresses N-terminal mCherry-tagged β-SNAP1 under control of the heat-shock promoter. We repeated heat shock treatment in *coa* mutants with transgene *Tg[hs:mCherry-β-SNAP1]* at a 12-h intervals from 36 hpf until either 60, 72, 84, 108, or 132 hpf (Fig. 4A). First, we evaluated cone survival by labeling with zpr1 antibody (Fig. 4A). Three heat-shock treatments at 36/48/60 hpf partially prevented cone apoptosis in *coa* mutants at 5 dpf, but did not at 8 dpf. Four treatments at 36/48/60/72 hpf prevented cone apoptosis in *coa* mutants until 8 dpf, but did not at 14 dpf. Interestingly, five treatments at 36/48/60/72/84 hpf prevented cone apoptosis until 14 dpf and even until 21 dpf in one of two cases (50%). Surprisingly, seven and nine heat-shock treatments from 36 until 108 and 132 hpf, respectively, stably prolonged cone survival period until 21 dpf in *coa* mutants, although the fraction of cone area in total retinal area was slightly but significantly reduced at 21 dpf (Fig. 4B, C). Since mCherry-β-SNAP1 protein disappears by 36 h after heat shock treatment (Fig. S2C), cones survive without β-SNAP1 activity after 6 dpf.

To evaluate rod survival, we introduced a zebrafish transgene *Tg[XlaRho:XP-GFP]* into wild-type and *coa* mutant lines carrying *Tg[hs:mCherry-β-SNAP1]*. Since rod OS genesis are markedly enhanced in the ventral retina in zebrafish^21,22^, we evaluated rod OS size by measuring XP-GFP positive area in the dorsal retina (Fig. 4B). In wild-type retinas with heat-shock treatment from 36 until 132 hpf, rod OS area progressively increased from 6 to 28 dpf (Fig. 4D). In *coa* mutants with heat-shock treatment from 36 until 132 hpf, rod OS area was similar to that of wild type at 6 dpf, but decreased after 10 dpf, and disappeared at 28 dpf (Fig. 4D, E). Thus, rods survived in *coa* mutants at 6 dpf when β-SNAP1 was overexpressed from 36 to 132 hpf, but degenerated after 6 dpf. Since the rod OS actively grows from 10 to 28 dpf (Fig. 2F), rod degeneration after 6 dpf is consistent with our model that BNip1-dependent photoreceptor apoptosis is linked to the OS growing period.

There are four *snap* genes, *α-snap1*, *α-snap2*, *β-snap1*, and *β-snap2*, in zebrafish, but only *β-snap1* mRNA is expressed in retinal photoreceptors during embryonic stages^2^. We confirmed that only *β-snap1* mRNA is expressed in retinal photoreceptors at 10 dpf and adult stages (Fig. S3A, S3B) and that *α-snap1*, *α-snap2*, and *β-snap2* are not upregulated in surviving photoreceptors of 19 dpf *coa* mutants with overexpression of β-SNAP1 from 36 to 108 hpf (Fig. S3C), excluding the possibility that α-SNAP1, α-SNAP2, or β-SNAP2 is ectopically expressed in photoreceptors at later stages in *coa* mutants in compensation for loss of β-SNAP1 and prevents cone photoreceptor apoptosis. Thus, cone photoreceptors survive after 6 dpf in the absence of SNAP activity in zebrafish. Taken together, these data suggest that BNip1-mediated photoreceptor apoptosis is specifically activated during the OS growth period.

### Müller cells are not reprogrammed for neuronal regeneration, but rod progenitors increase in 21 dpf-***coa*** mutants with overexpression of β-SNAP1 during the OS growth period

**Figure 5:**
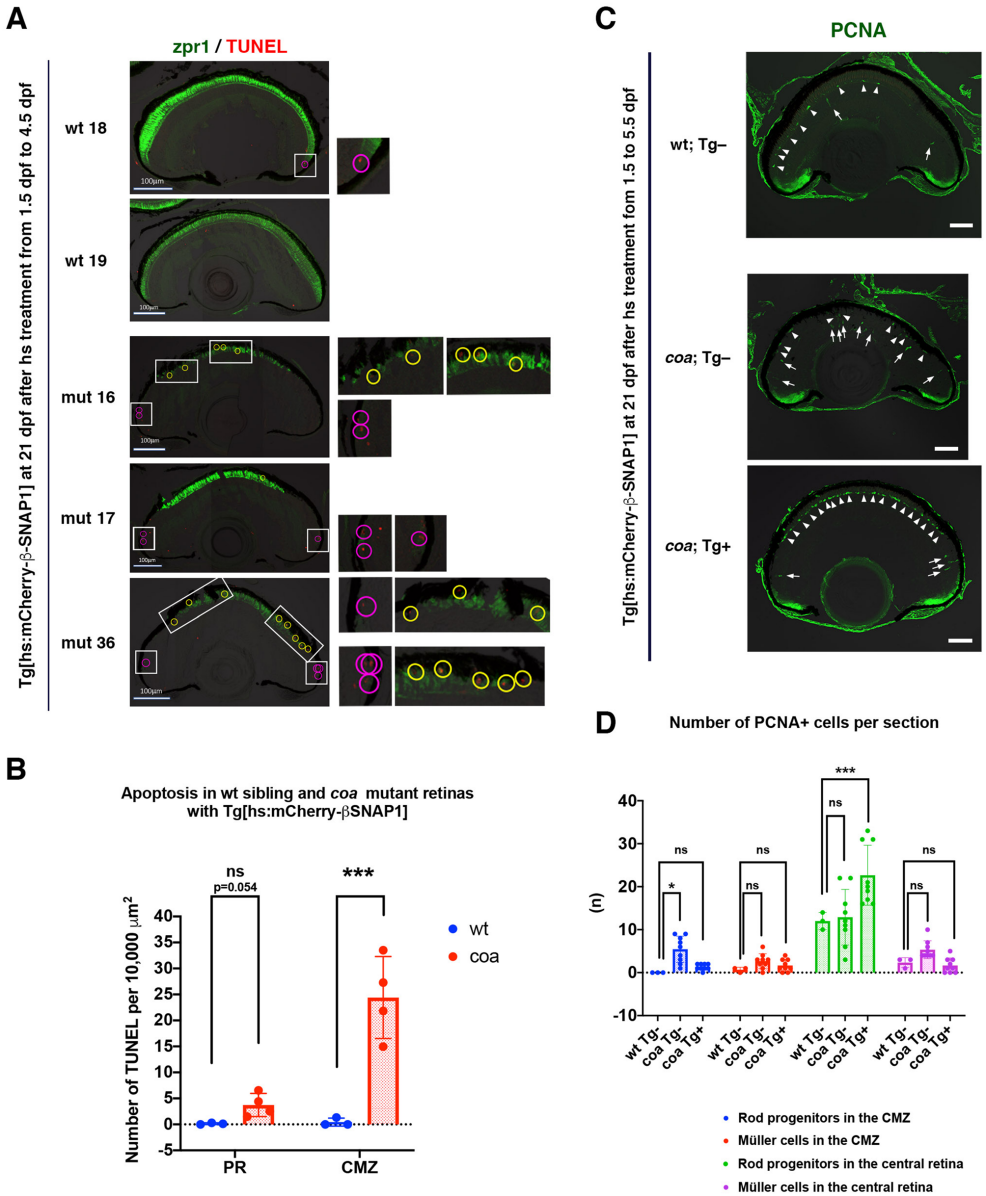
Photoreceptor apoptosis and neuronal regeneration in *coa* mutants with overexpression of β-SNAP1 during the OS growth period. (**A**) TUNEL of wild-type and *coa* mutant retinas with overexpression of β-SNAP1 during the initial OS growth period. Cones are visualized with zpr1 (green). Yellow and red circles indicate TUNEL signals in photoreceptors of central retinas and retinal CMZ, respectively. Higher magnification images of all TUNEL signals indicated by white squares are shown in the right side. Scale: 100 μm. (**B**) The number of TUNEL-positive cells per 10,000 μm^2^ in photoreceptors and CMZs of 21-dpf, wild-type siblings and *coa* mutants with overexpression of β-SNAP1 during the OS growth period. TUNEL density is slightly increased in rescued *coa* mutant photoreceptors, but does not differ significantly from that of wild-type siblings. On the other hand, TUNEL density in the CMZ is significantly higher in *coa* mutants than in wild-type siblings. Means ± SD. Two-way ANOVA with Sidak’s multiple comparison test. ***p < 0.005. (**C**) Anti-PCNA antibody labeling of wild-type and *coa* mutants with overexpression of β-SNAP1 during the initial OS growth period. Arrowheads and arrows indicate rod progenitor cells and reprogrammed proliferative Müller cells, respectively. Compared with wild-type control (upper), in *coa* mutants (middle), the number of reprogrammed proliferative Müller cells was increased in the central retina. In *coa* mutants with overexpression of β-SNAP1 during the initial OS growth period (bottom), the number of rod progenitor cells is markedly increased in the central retina; however, reprogrammed proliferative Müller cells were only observed in the peripheral retina and not in the central retina. Scale: 50 μm. (**D**) The number of PCNA-positive cells per section. In *coa* mutants with overexpression of β-SNAP1 during the initial OS growth period, numbers of proliferative Müller cells in both the CMZ (red bars) and the central retina (purple bars) were similar to that of wild type. Rod progenitors in the CMZ were not significantly different from those of wild type (blue bars). Interestingly, rod progenitors in the central retina were twice as numerous as in wild type (green bars). Means ± SD. Two-way ANOVA with the Tukey multiple comparison test. *p < 0.05.

Overexpression of β-SNAP1 from 36 to 108 hpf rescued cone survival in *coa* mutants until 21 dpf; however, rods continued to degenerate (Fig. 4C, E). In *coa* mutants with overexpression of β-SNAP1 from 36 to 108 hpf, apoptosis was observed in the surviving photoreceptor cell layer of the central retina as well as in the CMZ at 21 dpf, whereas apoptosis was rare in wild-type sibling retinas (Fig. 5A). However, the density of apoptotic cells in the surviving photoreceptor cell layer of *coa* mutants was slightly higher than that of wild type; however the difference was not significant (Fig. 5B). This suggests that apoptosis is effectively inhibited in the surviving photoreceptor cell layer in *coa* mutants with overexpression of β-SNAP1 during the initial OS growth period, although slightly increased apoptosis may be caused by ongoing degeneration of rods. In contrast, the density of apoptotic cells in the CMZ of *coa* mutants was significantly higher than that of wild-type siblings (Fig. 5B), suggesting that these CMZ photoreceptors are in the OS growing stage and undergo apoptosis.

In zebrafish, after photoreceptor damage, Müller cells are reprogrammed to assume a retinal progenitor cell state and to generate neuronal progenitor cells, which subsequently differentiate into all retinal cell types^23^. Rod progenitors normally produce rods after the embryonic stage throughout life^24,25^, and their proliferation is also activated in response to photoreceptor damage. Although apoptosis in the surviving photoreceptor cell layer was not significantly higher in *coa* mutants with overexpression of β-SNAP1 during the initial OS growth period (Fig. 5B), we examined whether a retinal regeneration program is activated. Labeling with anti-PCNA antibody can visualize both rod progenitor cells and proliferative reprogrammed Müller cells. In the central region of wild-type retinas at 21 dpf, the average number of rod progenitor cells and proliferative Müller cells were 12 and 2.33 per section, respectively (Fig. 5C, D). In *coa* mutants, the number of proliferative Müller cells increased in the central retina as well as the CMZ, although the difference was not significant. On the other hand, the number of CMZ rod progenitor cells was significantly higher than that of wild type; however, the number of central rod progenitor cells was similar to that of wild type (Fig. 5C, D). Interestingly, in *coa* mutants with overexpression of β-SNAP1 during the initial OS growth period, the number of rod progenitors increased twofold in the central retina, but was similar to that of wild type in the CMZ. Furthermore, the number of proliferative Müller cells was also similar to that of wild type in both the CMZ and the central retina (Fig. 5C, D). In summary, Müller cells are not reprogrammed for neuronal regeneration, but rod progenitors markedly increase in central retinas of *coa* mutants with overexpression of β-SNAP1 during the initial OS growth period. This is consistent with a subtle, but non-significant increase in apoptotic cell density in the surviving photoreceptor cell layer in *coa* mutants with overexpression of β-SNAP1 during the initial OS growth period at 21 dpf; however, this subtle increase of apoptotic cell density may trigger proliferation of rod progenitor cells.

### Reduction of vesicular transport from the ER to the OS suppresses photoreceptor apoptosis in *coa* mutants

**Figure 6:**
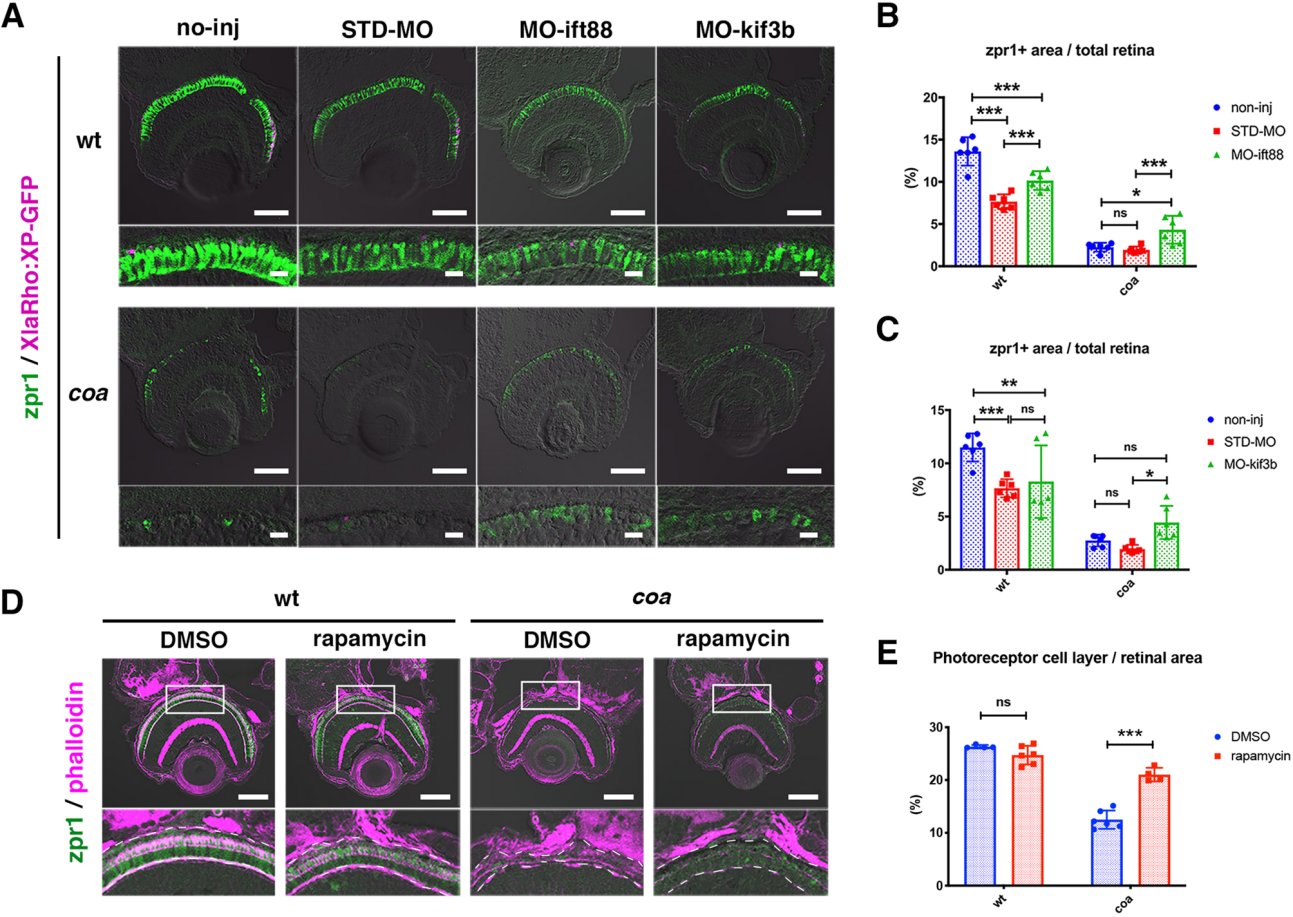
Blockade of intracellular vesicular transport rescues photoreceptor apoptosis in *coa* mutants. (**A**) Retinas of 3.5 dpf wild-type and *coa* mutant embryos injected with standard MO, MO-ift88, or MO-kif3b. Cone photoreceptors and rod OSs were visualized by labeling with zpr1 antibody (green) and fluorescent signals from *Tg[XlaRho:XP-GFP]* (magenta). In *coa* mutant retinas injected with either MO-ift88 or MO-kif3b, cone photoreceptor degeneration was partially inhibited. Scale: 50 μm (upper) and 10 μm (lower). (**B**) Percentage of zpr1-positive area relative to total retinal area in wild-type and *coa* mutant embryos injected with standard MO and MO-ift88. MO-itf88 significantly rescued photoreceptor apoptosis in *coa* mutants. Means ± SD. Two-way ANOVA with the Tukey multiple comparison test. *p < 0.05, ***p < 0.005. (**C**) Percentage of zpr1-positive area relative to total retinal area in wild-type and *coa* mutant embryos injected with standard MO and MO-kif3b. MO-kib3b significantly rescued photoreceptor apoptosis in *coa* mutants. Means ± SD. Two-way ANOVA with the Tukey multiple comparison test. *p < 0.05, **p < 0.01, ***p < 0.005. (**D**) Retinas of 3.5 dpf wild-type and *coa* mutant embryos treated with DMSO or rapamycin. Lower panels indicate higher magnification of squares in upper panels. The outline of the photoreceptor cell layer (white dotted lines) was visualized by labeling with zpr1 (green) and phalloidin (magenta). In *coa* mutant retinas treated with rapamycin, the size of photoreceptor cell layer increased. Scale: 50 μm. (**E**) Percentage of the photoreceptor cell layer relative to total retinal area in wild-type and *coa* mutant embryos treated with DMSO and rapamycin. Rapamycin treatment significantly recovers the size of photoreceptor cell layer in *coa* mutants. Means ± SD. Two-way ANOVA with the Tukey multiple comparison test. ***p < 0.005.

Next, to determine whether photoreceptor apoptosis in *coa* mutants is functionally linked to excessive vesicular transport, we examined whether photoreceptor apoptosis is rescued in *coa* mutants when vesicular transport from the ER to the OS decreases. In zebrafish and mice^26,27^, intracellular transport of photoreceptive proteins to the OS through the connecting cilium is mediated by Intraflagellar transport protein 88 (Ift88)^12^ and kinesin-2 family proteins, such as Kif3b^13^. In zebrafish *ift88* and *kif3b* mutants, the OS fails to form, but photoreceptors are still maintained at 96 hpf^12^ and 120 hpf^13^, respectively. We designed MO-ift88 and MO-kif3b in accordance with previous reports and used the same concentration. When MO-ift88 was injected into wild type, embryos showed a typical downward curled body shape linked to ciliary defects at 54 hpf (Fig. S4A). Alternative splicing was also inhibited, because MO-ift88 targets a splicing site (Fig. S4B). Third, photoreceptors do not undergo apoptosis at 84 hpf (Fig. S4C). Thus, MO-ift88 effectively inhibits ciliary transport functions, but photoreceptor degeneration phenotypes appeared later than 84 hpf. We inhibited functions of Ift88 and Kif3b by injection of morpholino antisense oligos (MO-ift88, MO-kif3b) and evaluated cone survival with zpr1 antibody labeling. MO-ift88 and MO-kif3b injection partially, but significantly, suppressed cone photoreceptor apoptosis in *coa* mutants at 84 hpf, compared with standard MO (STD-MO) (Fig. 6A–C).

Second, we treated wild-type and *coa* mutant embryos with rapamycin. Rapamycin inhibits the mTOR pathway, which promotes protein synthesis and transcription in response to nutrients and energy levels^14^. Rapamycin treatment generally inhibits protein synthesis, so zpr1 antibody signals would have been very weak. Thus, we evaluated photoreceptor survival with Rhodamine-conjugated phalloidin labelling. Phalloidin visualizes actin fibers associated with the outer plexiform layer (OPL), the outer limiting membrane (OLM), and connecting cilium, which outlines the photoreceptor cell layer. Rapamycin treatment from 36 to 96 hpf partially, but significantly, recovered photoreceptor survival in *coa* mutants (Fig. 6D, E). Since reduction of protein synthesis in the ER is likely to decrease vesicular transport to the OS, these data suggest that decreased vesicular transport from the ER to the OS suppresses photoreceptor apoptosis in *coa* mutants. These data support the possibility that excessive activation of vesicular transport activates BNip1 proapoptotic activity.

### The ER stress response is not activated in *coa* mutants during the OS growth period

**Figure 7:**
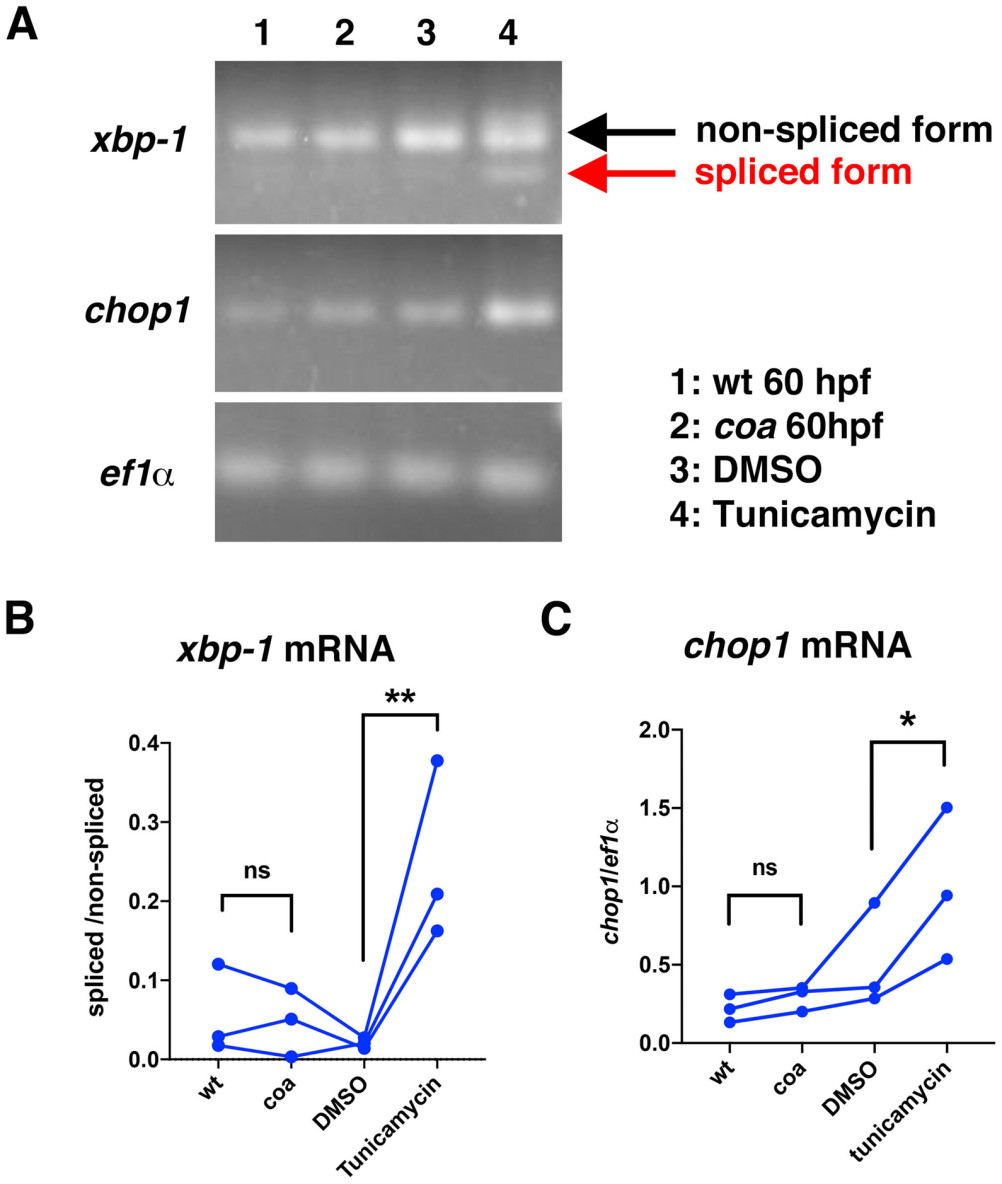
The ER stress response is not activated in *coa* mutants during the OS growth period. (**A**) Quantitative PCR of 60-hpf wild-type and *coa* mutant heads and 48-hpf wild-type embryos treated with DMSO and tunicamycin, using primers for the *xbp-1*, *chop1*, and *ef1α* genes. PCR amplified band images for *xbp-1*, *chop1*, and *ef1α* genes are cropped from the original full-length electrophoretic gel images shown in Fig. S5. The alternative spliced form of *xbp-1* and increased *chop1* mRNA expression were observed only in tunicamycin-treated embryos. (**B**) The ratio of the spliced form relative to the non-spliced form of *xbp-1* mRNA. We carried out three independent sets of PCR reactions for wild type, *coa* mutants, DMSO and Tunicamycin treatment. The same set of PCR reactions are connected with the line. The difference of *coa* mutant heads relative to wild-type heads, and Tunicamycin-relative to DMSO-treated embryos in *xbp1* spliced/non-spliced mRNA ratio were evaluated by Ratio paired t-test, two-tailed. p** < 0.01. There was no significant difference between wild type and *coa* mutants. (**C**) Ratio of *chop1* mRNA relative to *ef1α* mRNA. The difference of *coa* mutant heads relative to wild-type heads, and Tunicamycin-relative to DMSO-treated embryos in *chop1* mRNA expression were evaluated by Ratio paired t-test, two-tailed. p* < 0.05. There was no significant difference between wild type and *coa* mutants.

Depletion of β-SNAP activity compromises disassembly of cis-SNARE complexes after vesicular fusion, but eventually arrests recycling of SNARE molecules, which may activate the ER stress response. The ER stress response, also known as the unfolded protein response (UPR), restores ER homeostasis by increasing the protein-folding capacity in ER and decreasing protein load on the ER^11^. The ER stress response has three ER stress sensors: inositol-requiring protein-1α (IRE1α), activating transcription factor-6 (ATF6), and protein kinase RNA-like ER kinase (PERK). In response to ER stress, IRE1α promotes excision of a 26-nt intron of *xbp1* mRNA to generate a stable transcription factor XBPs^28^. PERK inhibits general protein translation through phosphorylation of eukaryotic translation initiator factor-2 (elF2α), but also activates selective translation of the transcription factor, ATF4, which subsequently contributes to induction of apoptosis through upregulation of CAAAT/enhancer-binding protein homologous protein (CHOP)^29^. Tunicamycin treatment induces the ER stress response, such as splicing of *xbp-1* and elevation of *chop1* mRNA expression (Fig. 7A). However, neither splicing of *xbp-1* nor elevation of *chop1* mRNA expression was observed in wild-type or *coa* mutant heads at 60 hpf (Fig. 7A-C). Thus, the ER stress response is not activated in *coa* mutants at the stage when BNip1-dependent apoptosis actively occurs. The BNip1-mediated apoptotic pathway is activated earlier than the ER stress response in zebrafish *coa* mutants.

## Discussion

**Figure 8:**
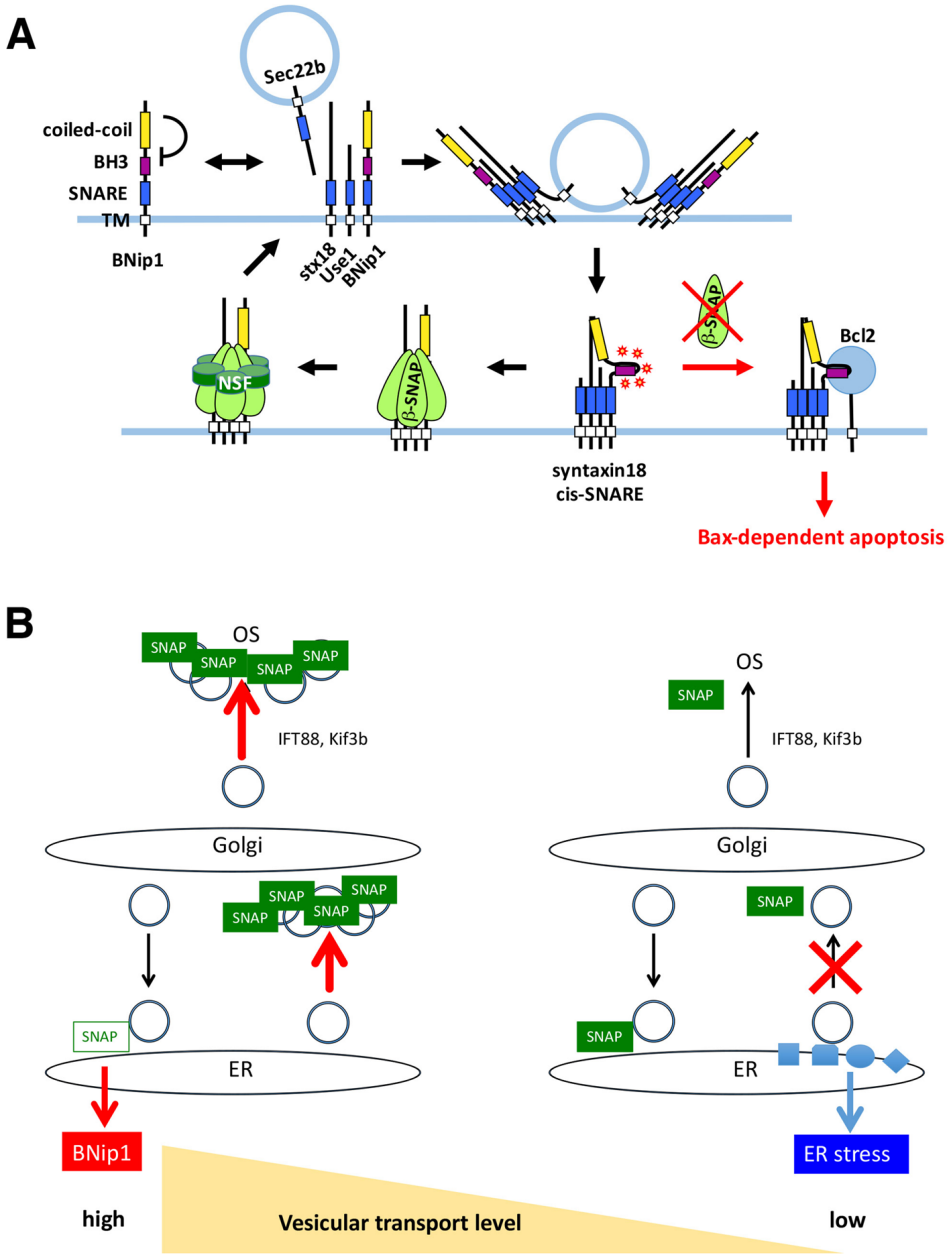
The role of BNip1-mediated apoptosis in photoreceptors. (**A**) The current model for molecular mechanism of BNip1-dependent apoptosis. In a monomer state, BNip1 proapoptotic activity is inhibited by intra-moleculer interaction between N-terminal coiled-coil domain and BH3 domain. After the vesicular fusion on ER membrane, BNip1 interacts with other components of the syntaxin-18 complex (Syntaxin18, Use1, Sec22b) through their SNARE domain and forms cis-SNARE complex, in which BNip1 BH3 domain is activated to interact with anti-apoptotic Bcl2, leading to the activation of Bax-dependent apoptosis. However, syntaxin-18 cis-SNARE complex is normally disassembled by SNAP and NSF, preventing BNip1 proapoptotic activity. In zebrafish *β-snap1* mutants, syntaxin-18 cis-SNARE complex is abnormally accumulated on ER membrane, which subsequently allows BNip1 to interact with Bcl2. (**B**) A model for physiological significance of BNip1-mediated apoptosis in photoreceptor differentiation and survival. When intracellular vesicular transport is arrested, synthesized proteins accumulate in the ER, subsequently triggering the ER stress response (Right panel). On the other hand, when intracellular vesicular transport is abnormally activated, vesicular fusion events increase on target membranes along the secretory pathway. Such excessive fusion events may trap SNAP molecules, which reduces the relative contribution of SNAP to vesicular fusion on the ER membrane. In this case, the syntaxin-18 cis-SNARE complex accumulates and the BNip1-dependent apoptotic pathway is activated (Left panel). Blockade of ciliary transport functions of Ift88 or Kib3b inhibits BNip1-dependent apoptosis, supporting this model. In this model, BNip1 may cooperate with the ER stress response to maintain appropriate levels of vesicular transport in photoreceptors.

Photoreceptors undergo BNip1-dependent apoptosis in zebrafish *β-snap1* mutants^2^. BNip1 is a t-SNARE component of the syntaxin-18 complex, which regulates retrograde transport from the Golgi to the ER. BNip1 also harbors a BH3 domain, which generally induces Bax-dependent apoptosis through an interaction with anti-apoptotic protein Bcl2. BNip1 monomer does not induce apoptosis, but co-expression of other components of the syntaxin-18 complex enhances BNip1 pro-apoptotic activity. β-SNAP1 normally promotes disassembly of the cis-SNARE complex, and the syntaxin-18 cis-SNARE complex accumulates abnormally in *β-snap1* mutants. From these observations, we proposed that BNip1 pro-apoptotic activity is activated through formation of the syntaxin-18 cis-SNARE complex (Fig. 8A). However, it is still a mystery what physiological conditions cause the syntaxin-18 cis-SNARE complex to accumulate in photoreceptors.

BNip1 is an ER-resident protein and the syntaxin-18 cis-SNARE complex is formed on the ER after vesicular fusion of retrograde-transported vesicles into the ER membrane. In this study, we examined whether BNip1 pro-apoptotic activity is activated on the ER membrane. Overexpression of ER-targeted Bcl2 effectively inhibited photoreceptor apoptosis in *β-snap1* mutants, implying that BNip1 pro-apoptotic activity is activated on the ER membrane. Next, to discover what physiological conditions induce BNip1-dependent apoptosis, we investigated the spatiotemporal profile of photoreceptor apoptosis in *β-snap1* mutants. We found that almost all photoreceptors, including both cones and rods, undergo apoptosis during a short developmental window from 60 to 96 hpf in *β-snap1* mutants. In zebrafish, cone differentiation starts at the ventronasal retina around 50 hpf and spreads to the entire retina by 72 hpf^30^. Recent cell lineage analysis of zebrafish retina revealed that cones are generated from 40 to 80 hpf^31,32^. On the other hand, rods begin to be generated at the ventronasal retina around 50 hpf, but their propagation to the dorsal and temporal retina proceeds until later stages than that of cones^30^. Thus, photoreceptor apoptosis occurs in the early stages of their differentiation. The most prominent feature during the early stages of photoreceptor differentiation is the formation of the OS. We examined the growth rate of cone and rod OSs. The cone OS grows rapidly from 3 to 5 dpf, reaching a plateau after 5 dpf. On the other hand, the rod OS initially grows until 4.5 dpf. Then its size is maintained at a plateau from 4.5 to 8.5 dpf, and it again starts to grow from 8.5 until 28 dpf, suggesting two growth phases. Consistently, it has been reported that the OS length in cones does not drastically increase after 8 dpf, whereas the rod OS rapidly increases in size between 12 and 20 dpf^19^. Thus, both cones and rods undergo apoptosis in *β-snap1* mutants during their initial OS growth period from 2 to 4 dpf. Although there is a second expansion phase of the rod OS, it is likely that the initial expansion of the rod OS is enough to trigger rod apoptosis in *β-snap1* mutants.

Next, to determine the critical period of β-SNAP activity for photoreceptor survival, we overexpressed β-SNAP1 during different time windows in *β-snap1* mutants using the heat shock promoter. Transient expression of β-SNAP1 from 36 to 132 hpf is enough to prevent cone and rod apoptosis in *coa* mutants at 6 dpf. Interestingly, cones continue to survive until 21 dpf. However, rods progressively degenerate after 6dpf. Since overexpressed β-SNAP1 protein can be maintained at least for 24 hpf after heat-shock treatment, it is likely that cones are maintained in the absence of β-SNAP activity after 6 dpf. On the other hand, rods failed to be maintained in the absence of β-SNAP after 6 dpf, probably because rod OS genesis is still active from 8.5 to 28 dpf. These data suggest that BNip1-dependent apoptosis is associated with OS growth of rod and cone photoreceptors.

Since protein and lipid synthesis increase during the OS growth period, their intracellular transport must be elevated during the OS growth period. Increased vesicular transport is likely to trap β-SNAP molecules on vesicular fusion sites, which subsequently decrease the relative contribution of β-SNAP to vesicular fusion events on the ER membrane, leading to accumulation of syntaxin-18 cis-SNARE complexes on the ER membrane (Fig. 8B). This scenario is consistent with our current model of the BNip1-mediated apoptosis mechanism. Indeed, ER-targeted Bcl2 effectively rescues photoreceptor apoptosis in *β-snap1* mutants, suggesting that BNip1 BH3 activation occurs on the ER membrane. Second, we did not observe activation of the ER stress response in *β-snap1* mutants at 2.5 dpf, suggesting that depletion of β-SNAP activity is detected by BNip1 prior to activation of the ER stress response. Third, we examined whether decreased intracellular transport rescues photoreceptor apoptosis in *β-snap1* mutants. Knockdown of ciliary transport regulators, Ift88 and Kif3b, partially, but significantly, rescues photoreceptor apoptosis in *β-snap1* mutants. Furthermore, rapamycin treatment, which inhibits protein synthesis through suppression of the mTOR pathway^14^ rescued photoreceptor apoptosis in *β-snap1* mutants. These observations suggest that BNip1 provides risk assessment to detect excessive activation of vesicular transport in photoreceptors. Since arrest of the anterograde transport pathway primarily causes retention of synthesized proteins in the ER and activates the ER stress response, BNip1 and the ER stress response may cooperatively determine an appropriate level of vesicular transport during photoreceptor development and homeostasis (Fig. 8B).

## Methods

### Ethics statement

All zebrafish experiments were performed in accordance with the Animal Care and Use Program of Okinawa Institute of Science and Technology Graduate School (OIST), which is based on the Guide for the Care and Use of Laboratory Animals by the National Research Council of the National Academies and has been accredited by the Association for Assessment and Accreditation of Laboratory Animal Care (AAALAC International). All experimental protocols were approved by the OIST Institutional Animal Care and Use Committee.

### Fish

Zebrafish (*Danio rerio*) were maintained according to standard procedures^33^. Okinawa wild type (*oki*) was used as a wild-type strain. *coa*^rw76b 2^ and *piy*^rw255 15^ mutant lines were used. Transgenic lines, *Tg[XlaRho:XP-GFP]*^oki04^, *Tg[hs:mCherry-β-SNAP1]*^oki07^, *Tg[hs:mCherry-tagged Bcl2]*^oki029^, *Tg[hs:mCherry-tagged Bcl2-ER]*^oki042^, *Tg[gnat2: GFP]*^oki061^^34^ were used.

### Histology

In situ hybridization, plastic sectioning, and immunolabeling of cryosections were performed as described previously^35^. Immunolabeling of whole retinas was conducted, using dissected optic cups of para-formaldehyde (PFA)-fixed embryos. zpr1 antibody (ZIRC, Eugene, Oregon; 1:100), anti-zebrafish green opsin (1:500)^20^, anti-red opsin (1D4, abcam ab5417, 1:200–500)^17^, anti-PCNA antibody (clone PC10, Sigma P8825; 1:200) were used. TUNEL was performed using an In Situ Cell Death Detection Kit (Roche). Rhodamine-conjugated phalloidin (Invitrogen, R415) at 0.66 μM was applied to visualize actin filaments on cryosections labelled with zpr1 antibody. Images were scanned using a confocal laser scanning microscope (Carl Zeiss, LSM510 and LSM710).

### Calculation of the zpr1-positive and cell death areas relative to total retinal area

The percentage of zpr1-positive area relative to total retinal area (Figs. 1D, E; 3B, C; 4B, C; 6A, B, C) was calculated using one cryosection image containing the central retina per eye, as previously described^2^. Means and standard deviations were calculated from data obtained in four or six retinal images from more than two embryos. Statistical analysis was done using two-way ANOVA with the Tukey multiple comparison test (Figs. 1E; 3C; 4C; 6B, C). The percentage of TUNEL-positive area relative to total retinal area (Fig. S1A, B) was calculated using cryosection images containing the central retina for each eye, as previously described^2^. Means and standard deviations were calculated from data obtained in four retinal images from more than three embryos. Statistical analysis was done using two-way ANOVA with the Tukey multiple comparison test (Fig. S1B).

The extent of the cell death area relative to the total retinal area (%) in *piy* mutants (Fig. S1C, D) was determined using one plastic section image containing the central retina for each eye. Using Image-J software (NIH), we demarcated the outline of the cell death area, which was represented by pyknotic nuclei, on each plastic section image. Then we compared its size with total retinal size. Means and standard deviations were calculated from data obtained for six retinal images from three embryos. Statistical analysis was done using one-way ANOVA with the Tukey multiple comparison test (Fig. S1D).

### Calculation of the XP-GFP-positive area relative to the total area of the photoreceptor cell layer

The percentage of the XP-GFP-positive area relative to total area of photoreceptor cell layer (Figs. 2E, F; 3B, D; 4D, E) was calculated in the dorsal retina using one cryosection image containing the central retina for each eye, similar to the method previously described^2^. Means and standard deviations were calculated. Statistical analysis was done using one-way ANOVA with the Dunnett’s multiple comparison test (Fig. 2F), and two-way ANOVA, the Sidak’s multiple comparison test (Fig. 3D, 4E).

### Calculation of TUNEL density in the photoreceptor cell layer and the CMZ

The number of TUNEL-positive cells was counted in the surviving photoreceptor cell layer or the CMZ of 19 dpf *coa* mutant and wild-type sibling embryos carrying the transgene *Tg[hs:mCherry-β-SNAP1]* with heat-shock treatment at 36/48/60/72/84/96/108 hpf (Fig. 5A). Three independent wild-type and four independent *coa* mutant retinas were used for TUNEL (red) and anti-zpr1 antibody labeling (green). The average number of TUNEL-positive cells was determined in the surviving photoreceptor cell layer and the CMZ, respectively, using 4 independent sections per individual retina. The zpr1-positive area and the CMZ area were outlined and their areas were determined using Image-J software (NIH). TUNEL signal density was calculated as the number of TUNEL per 10,000 μm^2^. Means and standard deviations were calculated. Statistical analysis was done using two-way ANOVA with Sidak’s multiple comparison test (Fig. 5B).

### Estimation of OS size during development

Using Image-J software (NIH), the OS size of individual cones was calculated from the outline of the green opsin-positive area on cryosection images of wild-type retinas labelled with anti-green opsin antibody at 3, 4, 5, 6, 7 and 8 dpf. The OS size of indivisual rods was calculated from the outline of the XP-GFP-positive area on cryosection images of wild-type retinas at 3.5, 4.5, 5.5, 6.5, 7.5, 8.5, 10, 15, 21 and 28 dpf. Means and standard deviations were calculated. Statistical analysis was performed using one-way ANOVA with the Tukey multiple comparison test (Fig. 2D) and the Dunnett’s multiple comparison test (Fig. 2F).

### Overexpression of β-SNAP1 in ***coa*** mutants

A transgenic line, *Tg[hs:mCherry-β-SNAP1]*, was established and combined with *coa* mutants. *coa*; *Tg[hs:mCherry-β-SNAP1]* embryos were maintained at 28.5 °C. Heat-shock treatment was accomplished by incubating these embryos at 39 °C for one hour between 36 and 132 hpf at 12-h intervals. As reported previously^2^, transient expression of mCherry-β-SNAP1 was done by injecting a DNA construct encoding *hs:mCherry-β-SNAP1* into one-cell-stage embryos. We also confirmed that mCherry-β-SNAP1 expression was stably detected at 60 and 72 hpf for heat-shock treatment at 48 hpf; at 72 and 84 hpf for heat-shock treatment at 60 hpf; at 84 hpf for heat-shock treatment at 72 hpf (Fig. S2A, S2C). Measurement procedures for mCherry-β-SNAP1 expression are shown in Fig. S2B.

### Generation of zebrafish transgenic lines expressing mCherry-tagged Bcl2 and mCherry-tagged Bcl2-ER

BNip1 is localized in the ER membrane via its C-terminal TM domain^2^. To design Bcl2-ER, we replaced the Bcl2 TM domain with a BNip1 TM domain. Furthermore, to visualize Bcl2 and Bcl2-ER, EGFP or mCherry was fused to their N-termini. To confirm that Bcl2-ER is localized in the ER, we injected mRNA encoding EGFP-Bcl2-ER (200 ng/μL) with mRNA encoding monomeric Kusabira Orange (mKO)-tagged ER retention peptides (ER-mKO) (200 ng/μL). We also injected mRNA encoding EGFP-Bcl2-ER (200 ng/μL) with mRNA encoding mKO-tagged mitochondrial-located peptides (MT-mKO) (400 ng/μL). We also generated DNA constructs expressing mCherry-Bcl2 and mCherry-Bcl2-ER under control of the heat shock promoter. We established zebrafish transgenic lines *Tg[hs:mCherry-tagged Bcl2]* and *Tg[hs:mCherry-tagged Bcl2-ER]*.

### Morpholino antisense-mediated knockdown of Ift88 and Kif3b

We used morpholino antisense oligonucleotides for Ift88 and Kif3b (GeneTools, LLC, Philomath, OR)^12,13^. Standard morpholino was used as a control. Morpholino was dissolved in water at 250 μM for MO-ift88, 500 μM for MO-kif3b and standard MO, and injected into wild-type embryos at the one-cell stage. MO sequences are shown below.

- MO-ift88, 5′-CAACTCCACTCACCCCATAAGCTGT-3’.
- MO-kif3b, 5′-AGCTCTTGCTTTTAGACATTTTGAC-3’.
- Standard MO, 5′-CCTCTTACCTCAGTTACAATTTATA-3’.

### Rapamycin treatment

In accordance with a previous report^36^, wild-type and *coa* mutant embryos were treated with rapamycin (LC laboratories, R-5000) at 10 μM from 34 to 97 hpf, and fixed in 4% PFA. Photoreceptor survival was evaluated with rhodamine-conjugated phalloidin labeling. Using Image-J software, the photoreceptor cell layer was outlined and its size was determined. Means and standard deviations were calculated. Statistical analysis was done using two-way ANOVA with the Tukey multiple comparison test.

### Assay of ER stress response

Total RNA was prepared from 2.5-dpf wild-type and *coa* mutant embryos. Complementary DNA strands were synthesized from total RNA using 3′RACE System for Rapid Amplification of cDNA Ends (Invitrogen, Cat#18373-027) and used as a template for semi-quantitative PCR. *xbp1*, *chop1*, and *ef1α* cDNAs were amplified with TaKaRa Ex Taq (TaKaRa Biochemicals), with 28 cycles. ER stress-induced wild-type samples were obtained by treatment with Tunicamycin (Nacalai code 35638-74, 2 μg/ml) from 25 to 48 hpf.

The following primers were used. Amounts were quantified using ImageJ (NIH).

- *xbp1* forward, 5′-GTTCAGGTACTGGAGTCCGC-3′.
- *xbp1* reverse, 5′-CTCAGAGTCTGCAGGGCCAG-3′.
- *chop1* forward, 5′-CAGAGAGCGCGAACAGGAGAATGAAAGG-3′.
- *chop1* reverse, 5′-CTGTTGCTCATTCACCTGCGGGTGTT-3′.
- *ef1α* forward, 5′-CGTGGTAATGTGGCTGGAGA-3′.
- *ef1α* reverse, 5′-CTGAGCGTTGAAGTTGGCAG-3′.

Means and standard deviations were calculated. Statistical analysis employed two-tailed Ratio paired t-tests.

## Supplementary information

Supplementary information.
