# Supplementary material for: β-SNAP activity in the outer segment growth period is critical for preventing BNip1-dependent apoptosis in zebrafish photoreceptors
Source: Sci Rep. 2020 Oct 15;10:17379. doi: 10.1038/s41598-020-74360-x (PMC7567113; doi:10.1038/s41598-020-74360-x)
Supplement: Supplementary file 1 — Supplementary information. [file 41598_2020_74360_MOESM1_ESM.docx]

**Supplementary Information**

**β-SNAP activity in the outer segment growth period is critical for preventing BNip1-dependent apoptosis in zebrafish photoreceptors**

**Yuko Nishiwaki, and Ichiro Masai**

**Supplementary Figures**

**Figure S1.** Bcl2-ER effectively rescues photoreceptor apoptosis in *coa* mutants

**Figure S2.** Evaluation of an experiment overexpressing mCherry-tagged β-SNAP1 with the heat shock promoter

**Figure S3.** *In situ* hybridization of *snap* genes in zebrafish retinas at 10 dpf and the adult stage

**Figure S4.** Photoreceptors do not undergo apoptosis by 84 hpf in zebrafish *ift88* morphants

**Figure S5.** Original electrophoretic gel images of quantitative PCR shown in Figure 7A

**
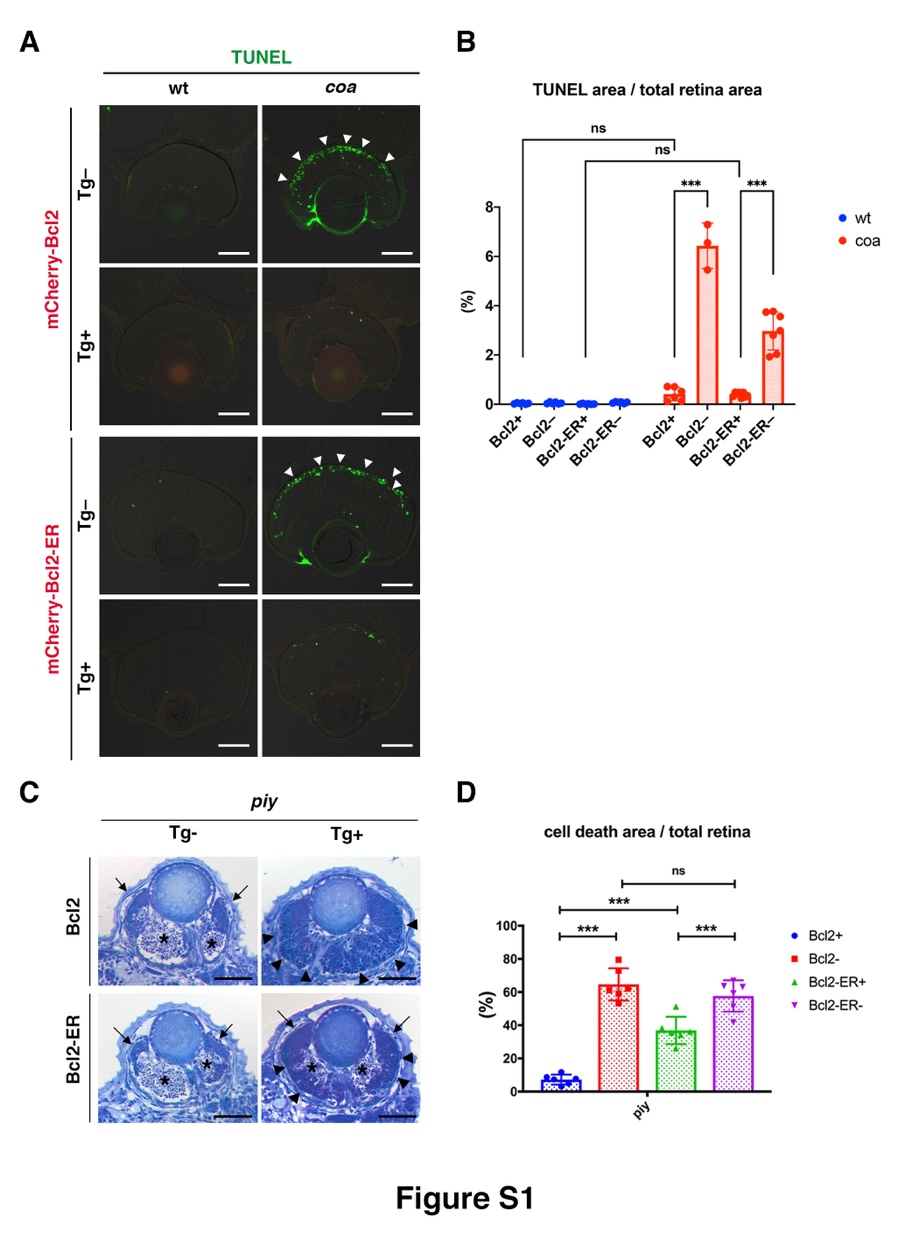
**

**Figure S1. Bcl2-ER effectively rescues photoreceptor apoptosis in *coa* mutants**

1. TUNEL of 3.5-dpf wild-type and *coa* mutant retinas combined with transgenic line *Tg[hs:mCherry-tagged Bcl2] or Tg[hs:mCherry-tagged Bcl2-ER]*. Tg+ and Tg- indicate transgenic and non-transgenic embryos, respectively. In *coa* mutants, severe apoptosis occurs in the photoreceptor layer (arrowheads). Both Bcl2 and Bcl2-ER rescue photoreceptor apoptosis in *coa* mutants. Scale: 50 μm.
2. Histogram of the percentage of TUNEL-positive area relative to total retinal area. Both Bcl2 and Bcl2-ER significantly inhibit photoreceptor apoptosis in *coa* mutants. Means±SD. One-way ANOVA with the Tukey multiple comparison test. ***p<0.005.
3. Three-dpf retinas of wild-type and *piy* mutant embryos combined with transgenic lines *Tg[hs:mCherry-tagged Bcl2]* or *Tg[hs:mCherry-tagged Bcl2-ER]*. Tg+ and Tg- indicate transgenic and non-transgenic embryos, respectively. In *piy* mutants, most retinal neurons undergo apoptosis (asterisks), although retinal stem cells are maintained and proliferate (arrows). Bcl2 effectively prevents retinal apoptosis in *piy* mutants (arrowheads). However, Bcl2-ER does not fully rescue retinal apoptosis in *piy* mutants (arrowheads), although the dying cell area diminishes (asterisks). Scale: 50 μm.
4. Histogram of percent cell death area relative to total retinal area. The cell death area occupies ~60% of the total retinal area in *piy* mutants. This percentage decreased to <10% in *piy*; *Tg[hs:mCherry-tagged Bcl2]* embryos with heat-shock treatment. However, the cell death area occupies around 40% of total retinal area in *piy*; *Tg[hs:mCherry-tagged Bcl2-ER]* embryos with heat-shock treatment. Means±SD. One-way ANOVA with the Tukey multiple comparison test. ***p<0.005.

**
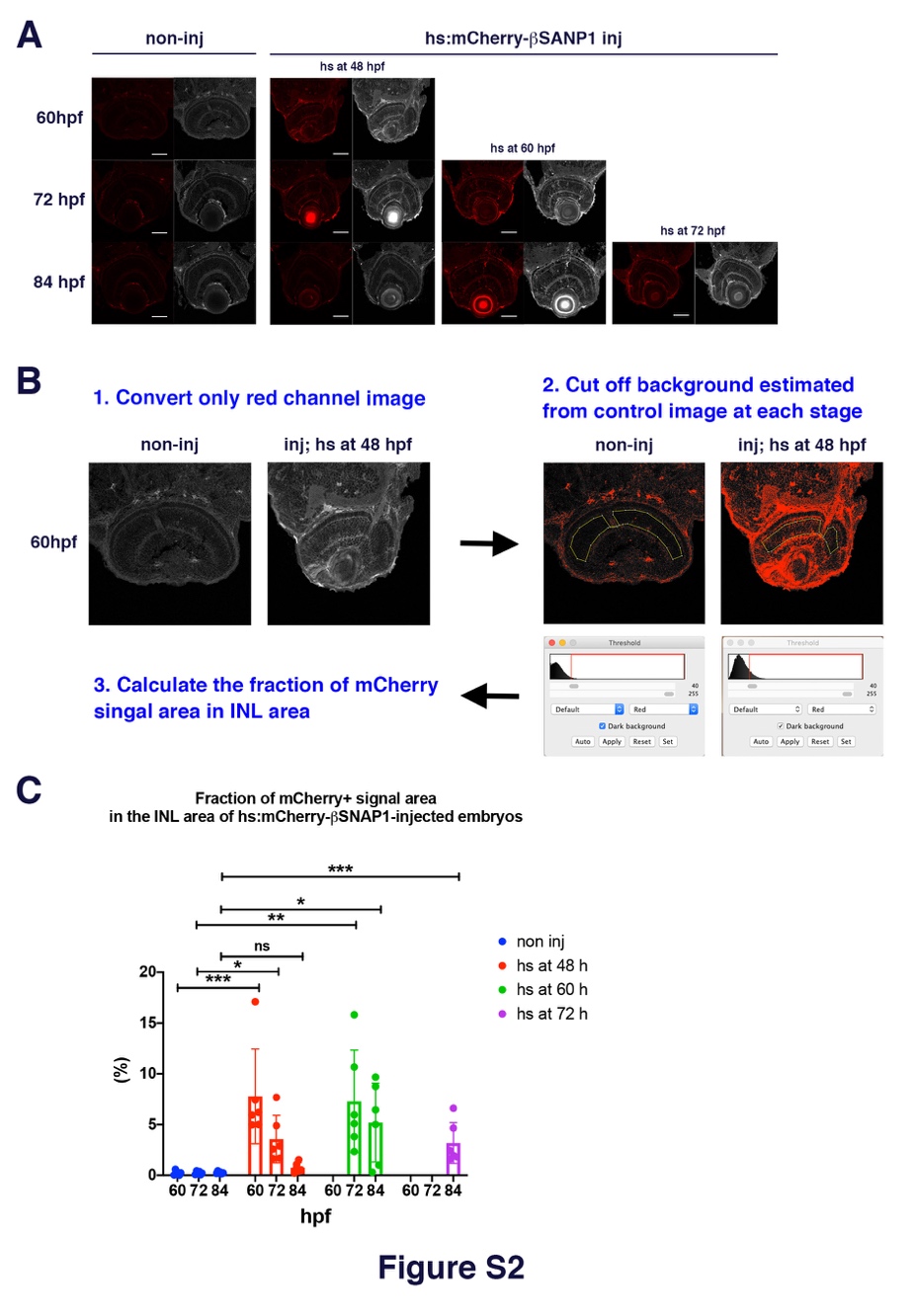
**

**Figure S2. Evaluation of an experiment overexpressing mCherry-tagged β-SNAP1 with the heat shock promoter**

1. Confocal scanning of mCherry fluorescence in the retinas of zebrafish embryos injected with the DNA construct encoding hs: mCherry-tagged β-SNAP1 under three different conditions: at 12, 24, and 36 hours after heat shock treatment at 48 hpf; at 12 and 24 hours after heat shock treatment at 60 hpf; at 12 hours after heat shock treatment at 72 hpf. Non injected embryos were used as a negative control. Right panels indicate only red channel images. Scale: 50 μm.
2. Experimental procedure evaluating the expression level of mCherry-tagged β-SNAP1 in each sample. To avoid auto-fluorescent noise, we focused on the inner nuclear layer (INL) area. After cutting off background noise estimated from control non-injected retinal images, the fraction of mCherry-signal area in the indicated INL area was calculated using image-J software.
3. Histogram of the fraction of mCherry-signal area in the INL area of hs: mCherry-tagged β-SNAP1-injected embryos. After heat shock treatment of injected embryos at 48 hpf, a significantly high level of mCherry-tagged β-SNAP1 expression was detected at 60 and 72 hpf, but not at 84 hpf. Similarly, mCherry-tagged β-SNAP1 expression is maintained at 72 and 84 hpf after heat shock treatment at 60 hpf, and at 84 hpf after heat shock at 72 hpf. These data indicate that mCherry-tagged β-SNAP1 expression is maintained at least 24 hpf after heat shock treatment.

**
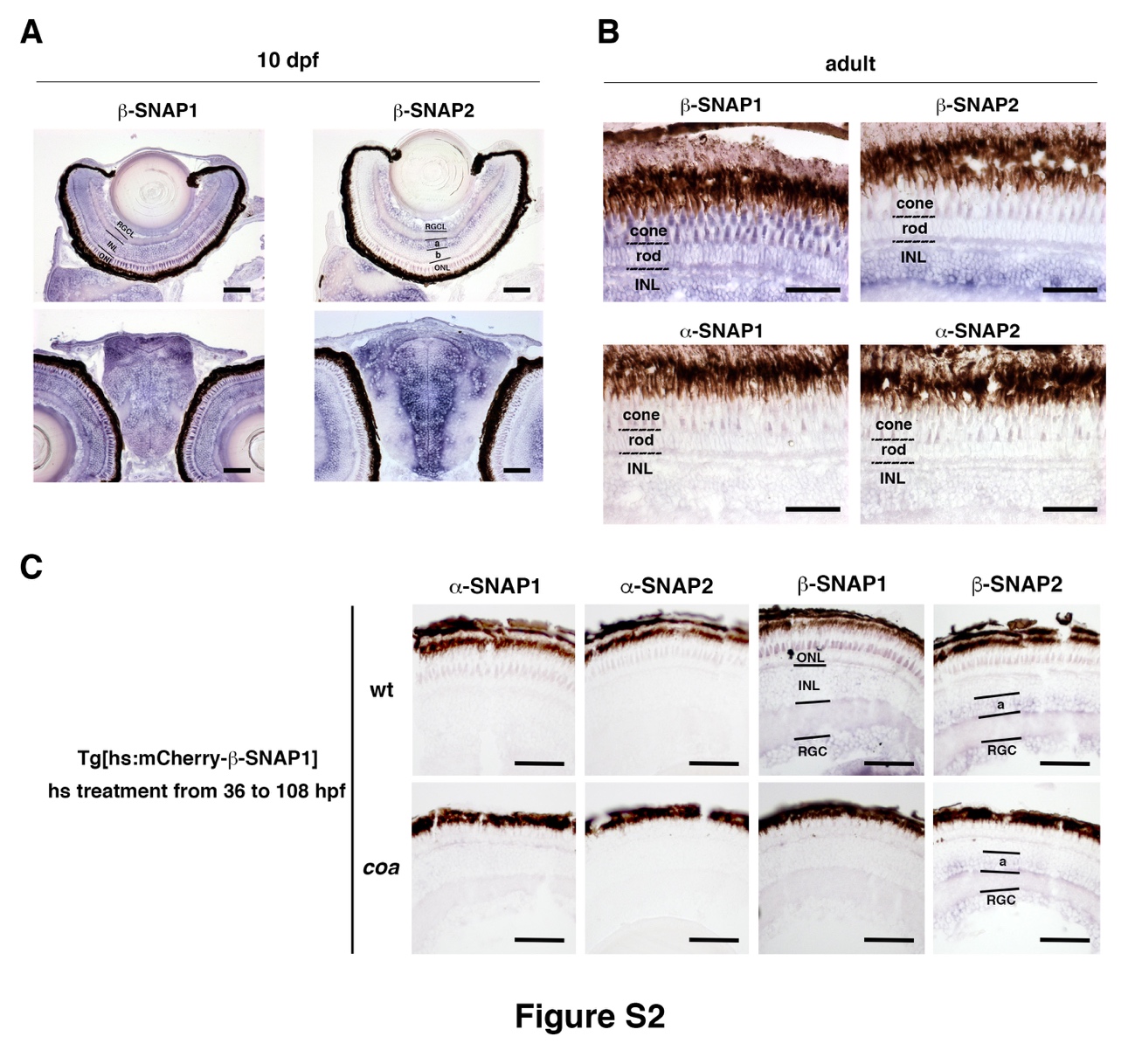
**

**Figure S3. *In situ* hybridization of *snap* genes in zebrafish retinas at 10 dpf and the adult stage**

1. Expression of *β-snap1* and *β-snap2* mRNA in 10-dpf retinas and forebrain. *β-snap1* mRNA is expressed in all retinal cell types. On the other hand, *β-snap2* mRNA is expressed in retinal ganglion cells and amacrine cells, but not in bipolar cells, horizontal cells, or photoreceptors. RGCL, retinal ganglion cell layer; a, amacrine cell layer; b, bipolar cell layer; INL, inner nuclear layer; ONL, outer nuclear layer. Scale: 50 μm.
2. Expression of *β-snap1*, *β-snap2*, *α-snap1*, and *α-snap2* mRNA in adult retina. *β-snap1* mRNA is expressed in all retinal cell types, including rod and cone photoreceptors, whereas *β-snap2* mRNA is expressed in retinal ganglion cells and the inner nuclear layer (INL), but not in cone and rod photoreceptors. *α-snap1*, and *α-snap2* mRNA are not expressed in the retina. cone, cone photoreceptor cell layer; rod, rod photoreceptor cell layer; INL, inner nuclear layer. Scales: 50 μm.
3. Expression of *β-snap1*, *β-snap2*, *α-snap1*, and *α-snap2* mRNA in 19-dpf wild-type and *coa* mutant retinas with overexpression of β-SNAP1 during the initial OS growing period. Expression of *snap genes* is similar to that of adults, whereas only *β-snap2* mRNA is expressed in RGCL and INL, but not in cone or rod photoreceptors in *coa* mutant retina. ONL, outer nuclear layer, INL, inner nuclear layer, RGC, retinal ganglion cell. Scale: 50 μm.


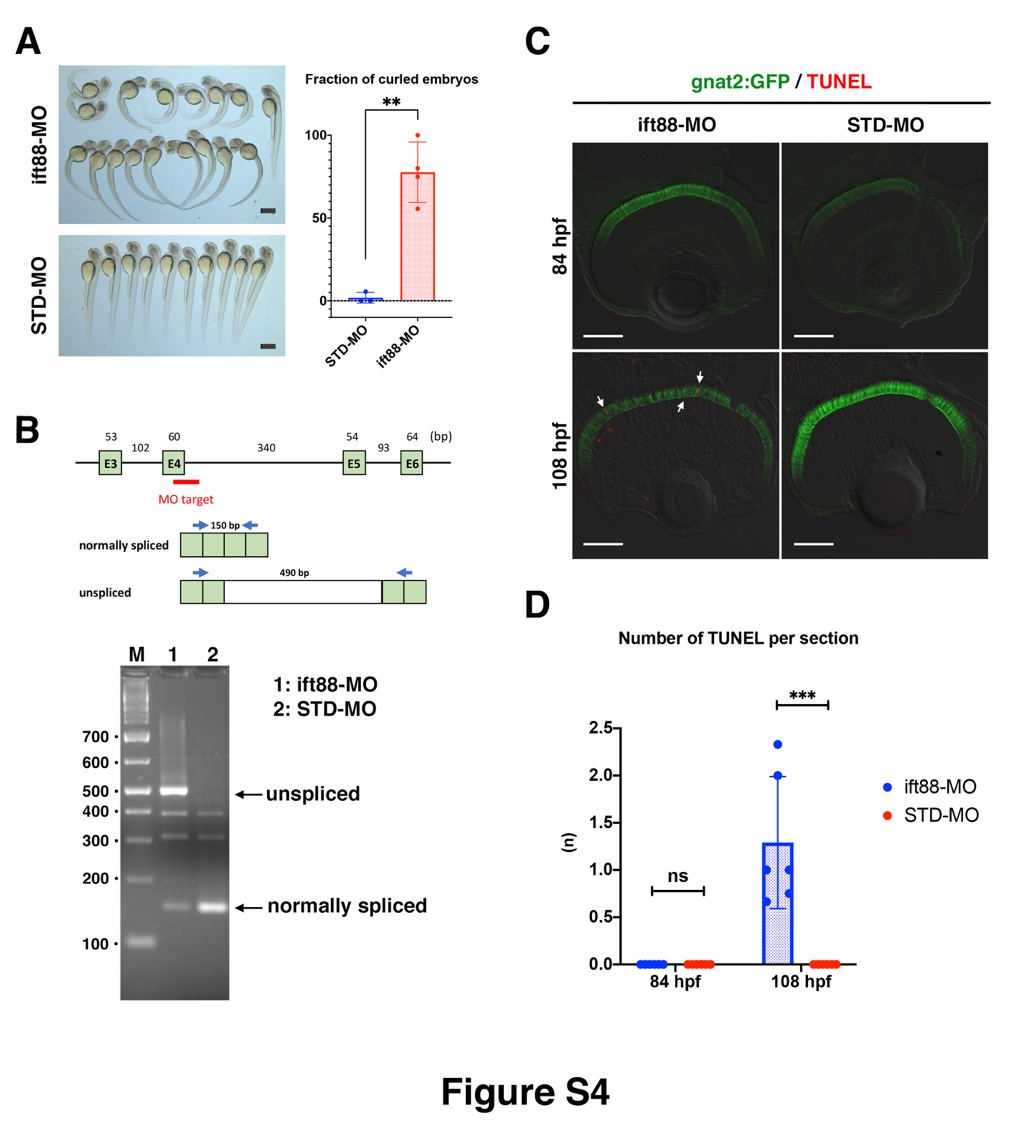


**Figure S4. Photoreceptors do not undergo apoptosis by 84 hpf in zebrafish *ift88* morphants**

1. Morphology of zebrafish embryos injected with *ift88*-MO (top left panel) and standard MO (bottom left panel) at 54 hpf. On average, 75 % of *ift88* morphants show a downward curled body axis, which is typical of cilia-defective mutants (right histogram). Means±SD. Unpaired t-test with Welch’s correction. **p<0.005. Scale: 500 μm.
2. *ift88*-MO used in this study interferes with alternative splicing of an intron between exon 4 and exon5, so we confirmed that this intron splicing is compromised in *ift88* morphants. Normally spliced mRNA (150 bp) was significantly reduced, whereas unspliced mRNA (490 bp) appeared in *ift88* morphants.
3. TUNEL of zebrafish embryos injected with *ift88*-MO and standard MO at 84 (top panels) and 108 hpf (bottom panels). Cone photoreceptors were counter-stained with the transgene *Tg[gnat2:GFP]*. No TUNUL signal was detected in *ift88* morphants at 84 hpf and standard MO-injected wild-type embryos at 84. A few TUNEL signals were detected in *ift88* morphants at 108 hpf (arrows). Scale: 50 μm.
4. Histogram of the number of TUNEL-positive photoreceptors per a retinal section. There was no significant difference between *ift88* morphants and standard MO-injected wild-type embryos at 84 hpf, suggesting that no apoptosis occurs in *ift88* morphant photoreceptors at 84 hpf. On the other hand, the TUNEL number was higher in *ift88* morphant photoreceptors than in standard MO-injected wild-type embryos at 108 hpf, although the number for each section was small (n=1.29), suggesting that photoreceptors start to undergo apoptosis at 108 hpf in *ift88* morphants. Means±SD. Two-way ANOVA with the Sidak’s multiple comparison test. ***p<0.001.


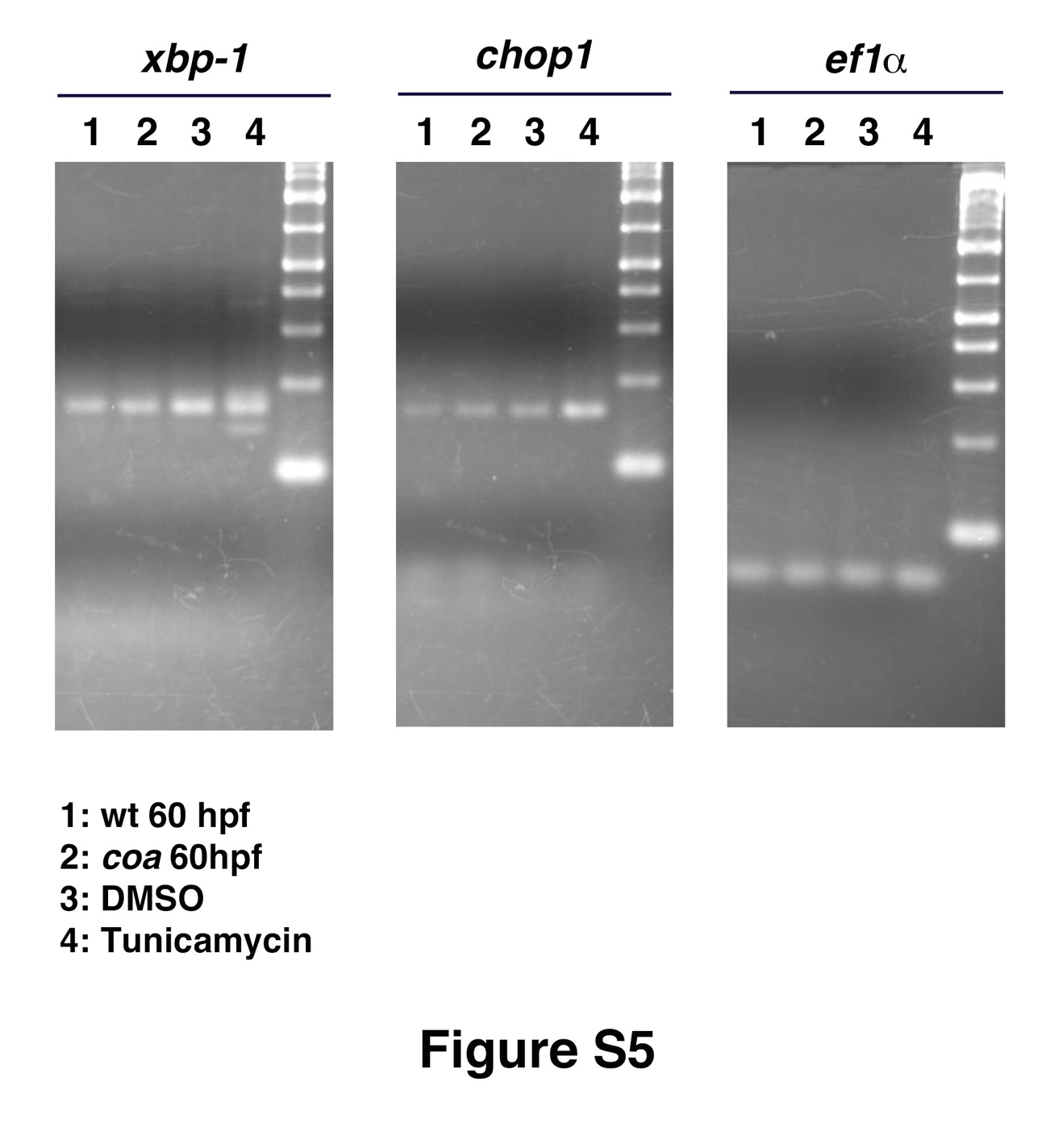


**Figure S5. Original electrophoretic gel images of quantitative PCR shown in Figure 7A**

Full-length gel images of quantitative PCR of 60-hpf wild-type and *coa* mutant heads and 48-hpf wild-type embryos treated with DMSO and tunicamycin, using primers for the *xbp-1*, *chop1*, and *ef1α* genes. Cropped images of PCR product bands are shown in Figure 7A. The right-most lane shows a DNA size marker, 100bp DNA ladder.
